# High-throughput quantum-mechanics/molecular-mechanics (ONIOM) macromolecular crystallographic refinement with *PHENIX*/*DivCon*: the impact of mixed Hamiltonian methods on ligand and protein structure

**DOI:** 10.1107/S2059798318012913

**Published:** 2018-10-29

**Authors:** Oleg Borbulevych, Roger I. Martin, Lance M. Westerhoff

**Affiliations:** a QuantumBio Inc., 2790 West College Avenue, State College, PA 16801, USA

**Keywords:** X-ray crystallography, quantum-mechanics refinement, PM6 semiempirical method, QM/MM, ONIOM macromolecular refinement, molecular mechanics, stereochemical restraints, ligand strain, *MolProbity* clashscore, high-throughput crystallography

## Abstract

Conventional macromolecular crystallographic refinement relies on often dubious stereochemical restraints, the preparation of which often requires human validation for unusual species, and on rudimentary energy functionals that are devoid of nonbonding effects owing to electrostatics, polarization, charge transfer or even hydrogen bonding. While this approach has served the crystallographic community for decades, as structure-based drug design/discovery (SBDD) has grown in prominence it has become clear that these conventional methods are less rigorous than they need to be in order to produce properly predictive protein–ligand models, and that the human intervention that is required to successfully treat ligands and other unusual chemistries found in SBDD often precludes high-throughput, automated refinement. Recently, plugins to the *Python-based Hierarchical ENvironment for Integrated Xtallography* (*PHENIX*) crystallographic platform have been developed to augment conventional methods with the *in situ* use of quantum mechanics (QM) applied to ligand(s) along with the surrounding active site(s) at each step of refinement [Borbulevych *et al.* (2014 ▸), *Acta Cryst* D**70**, 1233–1247]. This method (Region-QM) significantly increases the accuracy of the X-ray refinement process, and this approach is now used, coupled with experimental density, to accurately determine protonation states, binding modes, ring-flip states, water positions and so on. In the present work, this approach is expanded to include a more rigorous treatment of the entire structure, including the ligand(s), the associated active site(s) and the entire protein, using a fully automated, mixed quantum-mechanics/molecular-mechanics (QM/MM) Hamiltonian recently implemented in the *DivCon* package. This approach was validated through the automatic treatment of a population of 80 protein–ligand structures chosen from the Astex Diverse Set. Across the entire population, this method results in an average 3.5-fold reduction in ligand strain and a 4.5-fold improvement in *MolProbity* clashscore, as well as improvements in Ramachandran and rotamer outlier analyses. Overall, these results demonstrate that the use of a structure-wide QM/MM Hamiltonian exhibits improvements in the local structural chemistry of the ligand similar to Region-QM refinement but with significant improvements in the overall structure beyond the active site.

## Abstract 2

Quantum-mechanics/molecular-mechanics (ONIOM) X-ray macromolecular refinement using the program *DivCon* integrated with *PHENIX* is reported.

## 1. Introduction   

X-ray crystallography is a popular technique that is used to determine the three-dimensional atomic structures of bio­molecular systems, which serve as three-dimensional templates for structure-based drug discovery (SBDD) and fragment-based drug discovery (FBDD). The quality of the model is crucial for the overall success of high-throughput screening, docking and scoring (for example rank ordering) of potential drug candidates. In recent years, X-ray crystallography has become routine thanks to advances in data collection and processing, structure solution and refinement automation. However, protein crystal models are still subject to significant uncertainties in atomic coordinates and other structural errors (Davis *et al.*, 2003 ▸, 2007 ▸), and these errors negatively impact the very ligand-binding affinity estimations (Davis *et al.*, 2003 ▸) that are critical to SBDD/FBDD applications. This has led to the development of structure-validation metrics, including Ramachandran, clashscore and *MolProbity* score, the latter of which is a composite of the clashscore and Ramachandran plot and rotamer outliers (Ramachandran *et al.*, 2011 ▸; Read *et al.*, 2011 ▸; Chen *et al.*, 2010 ▸; MacCallum *et al.*, 2009 ▸). In particular, the median clashscore, which is the number of clashes per 1000 atoms, for all X-ray structures deposited in the Protein Data Bank (PDB) and the worldwide PDB (wwPDB) (Berman *et al.*, 2003 ▸, 2007 ▸) since 1990, and determined at a resolution of 1.5 Å or better, is 8.8 units. Furthermore, this median score deteriorates as the resolution decreases (Read *et al.*, 2011 ▸).

The prevalence of problematic geometries observed in deposited PDB structures suggests that conventional refinement methods are not sufficiently rigorous to represent the chemistry within the protein–ligand complex (Kleywegt, 2007 ▸; Pozharski *et al.*, 2013 ▸; Smart *et al.*, 2018 ▸). Overall, this problem stems from an intrinsic limitation of macromolecular X-ray crystallographic refinement, which is its reliance on an insufficient ratio of observed reflections to refined parameters, as typically observed at moderate and low resolutions (Rupp, 2009 ▸). In order to overcome this limitation, conventional refinement methods use *a priori* information about the structure in the form of stereochemical restraints (for example bond lengths, bond angles and bond torsion angles, as well as chirality and group planarity information) for all components included within the protein–ligand complex. For standard amino acids, these fixed stereochemical restraints are based on the ideal Engh and Huber parameters (Engh & Huber, 1991 ▸), and these restraints often lead to significant structural deficiencies (Moriarty *et al.*, 2014 ▸). In these situations, the backbone geometry can deviate significantly from these ideal values for high-resolution models (Vlassi *et al.*, 1998 ▸), and this problem becomes even more pronounced when small molecules and ions (for example, ligands, inhibitors and/or metallic or nonmetallic cofactors) are bound to the protein in question (Kleywegt, 2007 ▸). Surveys of the PDB indicate that the percentage of ligands with questionable geometric parameters in deposited macromolecular structures could be as high as 60% (Gore *et al.*, 2011 ▸; Liebeschuetz *et al.*, 2012 ▸).

These conventional methods rely on a detailed description of the molecular geometry for each species to be refined, and an accurate library or Crystallographic Information File (CIF) is important to the ultimate success of the effort. Unfortunately, the creation and validation of accurate CIFs is a non­trivial task which requires significant human intervention and often leads to bound ligand structures of less than desirable quality. These deficiencies are owing to the great variety of ligand chemistries and structures (Kleywegt, 2007 ▸), incomplete or inaccurate *a priori* understanding of *in situ* bound bond lengths and angles, and a lack of intermolecular interactions in conventional functionals (Read *et al.*, 2011 ▸). Efforts have been made in recent years to improve the automatic generation of ligand-restraint libraries for ligands in order to address these problems. The *eLBOW* tool (Moriarty *et al.*, 2009 ▸) found within the *Python-based Hierarchical Environment for Integrated Xtallography* (*PHENIX*) package (Adams *et al.*, 2010 ▸) is capable of creating restraints based on quantum-mechanics optimization, and the *AceDRG* tool from *CCP*4 provides similar capabilities (Nicholls, 2017 ▸; Long *et al.*, 2017 ▸). Alternatively, the publicly available *Grade* webserver (http://grade.globalphasing.org) along with the commercial *Mogul* package (Bruno *et al.*, 2004 ▸), which are both based on the Cambridge Structural Database (CSD; Groom *et al.*, 2016 ▸), use small-molecule X-ray structural information to determine target values. Finally, the *AFITT* program (Janowski *et al.*, 2016 ▸) produced by OpenEye Inc. works to improve the ligand geometry based on the Merck Molecular Mechanics Force Field (MMFF94). Regardless of the accuracy of the CIF, however, conventional methods are unable to accurately account for crucial binding influences on both the ligand and the surrounding active site arising from coordination, bond making/breaking, hydrogen bonding, electrostatics and other nonbonding interactions (Borbulevych *et al.*, 2014 ▸; Janowski *et al.*, 2016 ▸; Read *et al.*, 2011 ▸). This problem is further exacerbated when such species are covalently bound to the macromolecule.

Taking a different route, in 2014 our laboratory introduced (Borbulevych *et al.*, 2014 ▸) a plugin to the *PHENIX* package to treat the active site or the entire protein using our *DivCon* linear-scaling, semiempirical quantum-mechanics (SE-QM) implementation (Dixon & Merz, 1996 ▸, 1997 ▸) and the PM6 Hamiltonian (Stewart, 2009 ▸; Řezáč *et al.*, 2009 ▸). The advantage of this approach is that interactions such as hydrogen bonding, dispersion, electrostatics, polarization and charge transfer between the ligand and the protein are taken into account (Diller *et al.*, 2010 ▸; Zhang *et al.*, 2010 ▸). While the *DivCon* implementation can be applied to structures with thousands or even tens of thousands of atoms, the plugin was designed to optionally focus the QM method on one or more user-definable regions (for example active sites, ligands, key residues *etc.*) during the refinement (Region-QM), leaving the rest of the macromolecule dependent on conventional stereochemical restraints. In the present work, we explore a ‘complete functional’ representation for macromolecular refinement which uses a mixed quantum-mechanics/molecular-mechanics (QM/MM) Hamiltonian based on the ONIOM (Our own *N*-layered Integrated molecular Orbital and molecular Mechanics) method (Vreven *et al.*, 2003 ▸) as recently implemented in *DivCon Discovery Suite* build-7.1.1-b4015.17 (QuantumBio, 2017 ▸). We use SE-QM for the high-level theory, ‘region layer’ [including ligands(s) and active site(s)], while the remainder of the biomolecule, called the ‘system layer’, is treated using our implementation of the Assisted Model Building with Energy Refinement (AMBER) molecular-mechanics force field (Case *et al.*, 2014 ▸). In addition to validating the ONIOM refinement method against our previous Region-QM method, the results of conventional refinement as provided by the *PHENIX* platform are also discussed.

## 2. Methods   

### 2.1. *PHENIX* refinement and the QM/MM methodology   

Typical biomacromolecular systems, such as those including protein, DNA and/or RNA, are usually quite large and *ab initio* or density functional theory (DFT) QM methods are too expensive to treat these structures quickly and efficiently on the timescales demanded by industrial practitioners. The *DivCon Discovery Suite* (QuantumBio, 2017 ▸) employs divide-and-conquer (D&C), linear scaling, semiempirical quantum-mechanics (SE-QM) methods described previously (Dixon & Merz, 1996 ▸, 1997 ▸; Van der Vaart, Gogonea *et al.*, 2000 ▸; Van der Vaart, Suarez *et al.*, 2000 ▸; Wang *et al.*, 2007 ▸) to characterize all-atom structures of tens or even hundreds of thousands of atoms using the traditional AM1 (Dewar *et al.*, 1985 ▸) or PM3 (Stewart, 1989 ▸) SE-QM Hamiltonians, as well as the more modern PM6 Hamiltonian (Stewart, 2009 ▸; Řezáč *et al.*, 2009 ▸). Over the last two decades, this approach has been applied to a number of key SBDD applications including QMScore (Diller *et al.*, 2010 ▸; Merz & Raha, 2011 ▸; Raha & Merz, 2005 ▸; Zhang *et al.*, 2010 ▸) and NMRScore (Wang *et al.*, 2004 ▸, 2007 ▸; Williams *et al.*, 2009 ▸), QM-based quantitative structure–activity relationship (QSAR) models (Dixon *et al.*, 2005 ▸; Peters & Merz, 2006 ▸; Zhang *et al.*, 2010 ▸) and X-ray refinement (Borbulevych *et al.*, 2014 ▸, 2016 ▸; Li *et al.*, 2010 ▸; Yu *et al.*, 2005 ▸).

While the *DivCon* D&C implementation is faster than conventional semiempirical implementations, density functional theory (DFT) and *ab initio* QM methods (Dixon & Merz, 1996 ▸, 1997 ▸), linear-scaling SE-QM methods can still be time-consuming for large biomacromolecular structures (especially within an industrial environment, where a quicker turnaround time is often required). Therefore, the mixed QM/MM Hamiltonian concept provides a reasonable tradeoff for these structures as it allows one to treat the region of interest, such as an active site, at an SE-QM level of theory, while the remaining residues outside this region are treated at a faster, more approximate molecular-mechanics (MM) level of theory. This approach combines these different levels of theory in a way which significantly improves the speed of the calculation *versus* treating the entire structure at the higher level, but with a greater accuracy than if the entire structure were treated at the lower level (Chung *et al.*, 2015 ▸).

There are generally two QM/MM coupling schemes in common use in the computational chemistry field today: additive (Liu *et al.*, 2014 ▸) and subtractive (Vreven *et al.*, 2003 ▸). Additive QM/MM represents the energy of the system as the sum of three terms,
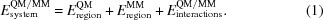
The first two terms describe the energies of the QM and MM regions, respectively, and the third term explicitly expresses interactions (coupling) between the QM and MM subsystems in the form of an additional, one-electron QM Hamiltonian describing the electrostatic coupling interactions between the two layers (Brooks *et al.*, 1983 ▸; Field *et al.*, 1990 ▸). This coupling term leads to greater complexity in the Hamiltonian, and calculating this term accurately can be particularly difficult given the inclusion of link atoms and electrostatic perturbations in the QM Hamiltonian (Plotnikov *et al.*, 2011 ▸).

Subtractive QM/MM, on the other hand, represents the energy of a system through the following equation (Vreven *et al.*, 2003 ▸),
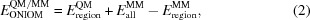
where the 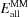 term is the MM energy calculated for the entire system, the 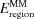 term is the MM energy for a region and 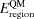 is the energy of the region computed using the QM method. As per Vreven *et al.* (2003 ▸), QM/MM ONIOM gradients in the subtractive scheme are computed using (3), which is similar to (2),
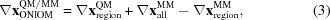
and in which the gradients of the QM region(s) include contributions from both the QM and the MM functionals. While standard ONIOM does not include electrostatic perturbations of the QM density matrix by the atoms within the MM region, the lack of a coupling term representing the inter­actions between these two regions in subtractive QM/MM leads to generally faster and more convergent calculations, along with the ability to treat multiple QM regions (such as those with multiple active sites or sites of interest or those with multiple copies). This makes the method particularly well suited to fast, routine, high-throughput QM/MM-based crystallographic refinement. With the use of the gradients represented in (3) [Disp-formula fd3], which utilize both QM and MM terms, we can approximate the interactions between the QM region and the MM region in a way that does not adversely impact on the convergence of the QM calculation.

**Figure 1:**
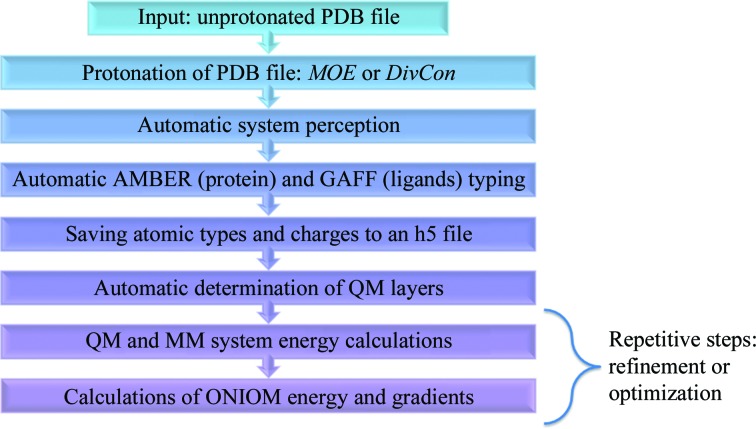
A flowchart of protein–ligand file treatment in ONIOM calculations.

Traditionally, with the explosion of different approaches and implementations, both general QM/MM varieties are often difficult to use depending upon the application and the desired outcomes of the investigator (Sousa *et al.*, 2016 ▸; Cao & Ryde, 2018 ▸). They can exhibit problems with convergence and performance which make the routine use of the methods expensive (Hu *et al.*, 2011 ▸), they are often limited to a single, compact QM region (Case *et al.*, 2018 ▸), they require significant atom-type and charge preparation of any unknown species (for example ligands, cofactors, nonstandard amino acids *etc.*) and protonation (Chung *et al.*, 2015 ▸), and/or they rely on the ability of a user to correctly define the QM atoms/residues along with any link atoms needed to complete broken bonds (Sousa *et al.*, 2016 ▸). As depicted in Fig. 1 ▸, the QM/MM implementation in *DivCon* addresses these problems through the inclusion of the following key features.

(i) The pervasive use of modern, QM energy-convergence algorithms.
(ii) Automatic perception and characterization of ‘unknown species’ (for example ligands, cofactors and ions) along with any closed-shell metal ions supported by our implementation of the PM6 SE-QM Hamiltonian (Stewart, 2009 ▸; Řezáč *et al.*, 2009 ▸).
(iii) Integrated protonation methods which include effects owing to pH, hydrogen bonding, clashes and ring-flip states.
(iv) Support for multiple QM region(s) through automatic residue-based selection, expansion and broken-bond completion.
(v) Automatic typing of crystallographically truncated residues and covalently bound residues and ligands.

**Figure 2:**
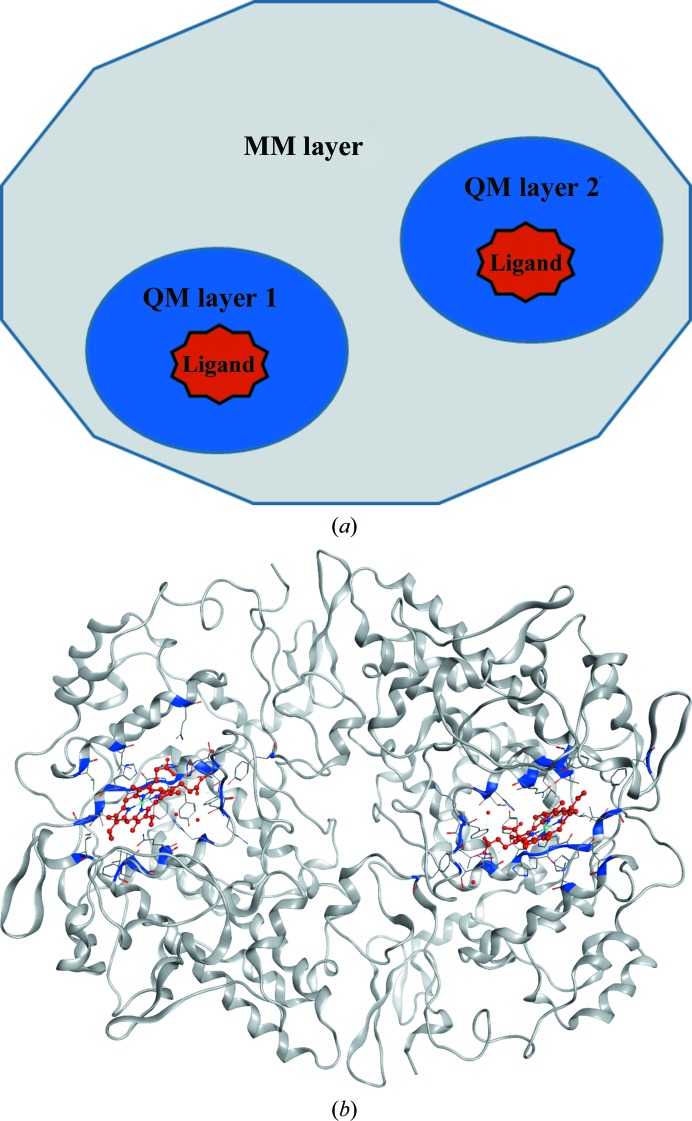
(*a*) Schematic view of the ONIOM two-layer (MM/QM) concept). (*b*) A PDB structure with two ligand regions to illustrate the ONIOM refinement concept.

The *DivCon Discovery Suite* build-7.1.1-b4015.17 was used for all QM/MM (ONIOM) calculations in this project. This package includes implementations of the SE-QM Hamilton­ians AM1 (Dewar *et al.*, 1985 ▸), PM3 (Stewart, 1989 ▸) and PM6 (Stewart, 2009 ▸; Řezáč *et al.*, 2009 ▸) along with an implementation of the AMBER MM force field (Case *et al.*, 2014 ▸). In the present project, we employed a two-layer ONIOM configuration as depicted in Figs. 2 ▸(*a*) and 2 ▸(*b*) where, for each characterized structure, the ligand(s) along with the surrounding active site was (were) treated using the PM6 SE-QM Hamiltonian and the remainder of the protein was treated using the 2014 parameter set of the AMBER MM force field. Both PM6 and AMBERFF14 were chosen as they are the most advanced methods available in the *DivCon Discovery Suite* at this time and they include a large coverage of atoms and atom types (for example, PM6 includes support for upwards of 70 elements). Furthermore, while newer SE-QM methods are available in the literature in other packages, such as PM7 (Stewart, 2013 ▸), recent benchmarks indicate similar performance characteristics between PM6 and PM7, with PM6 often demonstrating superior results (Hostaš *et al.*, 2013 ▸). Given these observations, the impact of the choice of SE-QM Hamiltonian on the results observed in the present study would be negligible.

Building on the QM-based plugin that we described in detail in Borbulevych *et al.* (2014 ▸), the ONIOM QM/MM method was integrated with the *PHENIX* package v.1.11.1-2575 (Adams *et al.*, 2010 ▸). The typical refinement protocol in* PHENIX* involves fitting bulk-solvent parameters and anisotropic scaling, reciprocal-space atomic coordinate refinement, atomic displacement parameter (ADP) refinement and occupancy refinement. The overall refinement target *E*_total_ in *PHENIX* is presented as
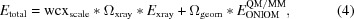
where Ω_xray_ and Ω_geom_ are weights assigned to X-ray data and geometry (QM/MM ONIOM) restraints, respectively, and wcx_scale_ is an additional scale factor implemented in *PHENIX* (Afonine *et al.*, 2012 ▸). Ω_geom_ is typically set to 1, while Ω_xray_ is a variable weight determined using an automatic procedure in *PHENIX* (Adams *et al.*, 1997 ▸). Mimicking the Region-QM refinement framework detailed in Borbulevych *et al.* (2014 ▸), (4) is extended in order to calculate the ONIOM QM/MM gradients on each atom with coordinates **x** according to
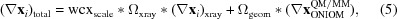
where 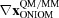 corresponds to the ONIOM gradients determined using (3), where any ligands and surrounding binding pockets is are defined as part of the QM region and the remainder of the structure is designated as the MM region. Under this regime, unlike in our prior work, all stereochemical restraint gradients are replaced by QM/MM gradients.

### 2.2. Structure preparation and refinement   

Coordinates and structure factors for all 80 structures from the Astex Diverse Set (Hartshorn *et al.*, 2007 ▸; Table 1 ▸, Supplementary Table S1) were downloaded from the PDB. Ligands(s), solvent molecules, metals and/or anions (*e.g.* Cl^−^) were included in each of the refinements. Since QM/MM is an ‘all-atom’ method (requiring protons as well as heavy atoms), H atoms were added to each structure, including all water molecules, using *Protonate*3*D* (Labute, 2009 ▸) as implemented in *MOE*2016 from Chemical Computing Group Inc. Likewise, CIFs for any unsupported species were automatically generated using Scientific Vector Language (SVL) extensions to *MOE*2016 provided in the *DivCon Discovery Suite*. For each structure in the set, every copy of each ligand specified in Table 1 ▸ was chosen as one or more QM region centers. The QM region(s) of each structure was (were) extended 3.0 Å from each center to include all amino-acid residues, ions and crystal waters within each pocket. The balance of residues and crystal waters were defined as part of the MM region and capping link atoms were automatically added to the QM region edges to satisfy covalent bonds that were cut in the process. In order to compare the new QM/MM refinement with older methods, we also refined the structures using both conventional (*i.e.* non-QM *PHENIX*) refinement and the Region-QM approach as described in our previous work (Borbulevych *et al.*, 2014 ▸). The same input PDB files were used in all three types of refinement, and in order to characterize automated refinement, only default parameters and automatically determined X-ray weights (Adams *et al.*, 1997 ▸, 2010 ▸) were used for *phenix.refine*. The aforementioned CIF files were used in the conventional refinement and they were provided as input to *PHENIX* in the Region-QM and ONIOM refinements in order to satisfy the internal ‘error-trapping’ mechanism of the *phenix.refine* executable. Certainly, we could spend a significant amount of time manually manipulating the input parameters, weights and restraints in order to ‘tune’ the conventional refinement for each of the 80 structures of the Astex Diverse Set; however, this approach could arguably no longer be considered high-throughput. Furthermore, from a scientific perspective, with too much ‘hand manipulation’ one would need to ask how much investigator bias could be introduced into the final model. Therefore, the approach utilized in the present study works to minimize investigator bias so that the final models are based solely on the combination of the experimental data and the initial placement of each structure as published, along with the Hamiltonian used for the refinement.

### 2.3. Validation metrics   

In order to validate the performance of ONIOM refinement in comparison to other refinement types (conventional and Region-QM), we employed two groups of metrics: ligand quality, consisting of both the strain energy and *Z* score of the difference density (ZDD; Tickle, 2012 ▸) assessed using *DivCon* (Borbulevych *et al.*, 2014 ▸; QuantumBio, 2017 ▸), and overall structure quality including *MolProbity* metrics assessed using the *MolProbity* program (Chen *et al.*, 2010 ▸) as distributed within the *PHENIX* package.

**Figure 3:**
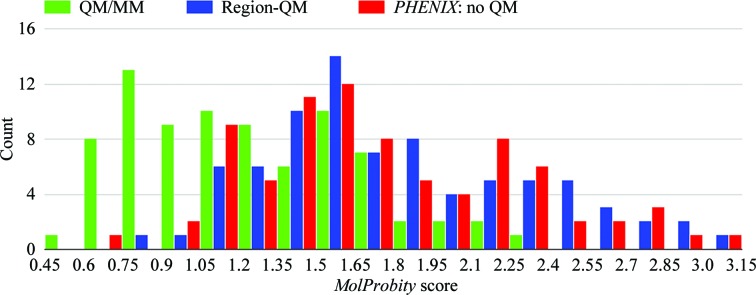
Histogram of MPScore distributions for 80 Astex structures refined with three methods: ONIOM, Region-QM and conventional.

**Figure 4:**
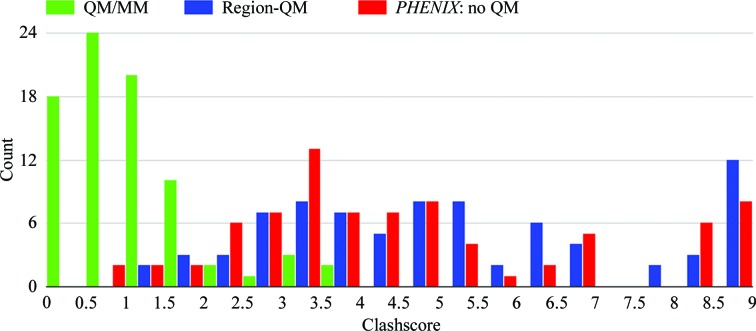
Histogram of *MolProbity* clashscore distributions for 80 Astex structures refined with three methods: ONIOM, Region-QM and conventional.

Since the histograms depicted in the present study (Figs. 3, 4, 6 and 9) show that the results are skewed and deviate from the normal distribution, instead of standard deviations (SDs) to show the spread of the data in the sections below, we employed the median absolute deviation (MAD; Sachs, 1984 ▸) calculated as
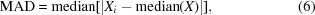
where *X_i_* represents data point *i* and *X* is the array of data.

#### 2.3.1. Local ligand-strain energy calculations   

Local ligand-strain energy is the difference in the conformational energy of the isolated ligand conformation and the protein-bound ligand conformation. This metric serves as a quality indicator of protein–ligand structures as it shows how much strain the ligand must take on or ‘accept’ in order to bind to the protein, and lower strain energy is preferred to higher strain energy (Fu *et al.*, 2011 ▸; Janowski *et al.*, 2016 ▸; Mobley & Dill, 2009 ▸; Perola & Charifson, 2004 ▸). Previously, we used ligand strain to validate Region-QM refinement and we validated the method against a repertoire of 50 quasi-randomly chosen PDB structures (Borbulevych *et al.*, 2014 ▸); we went on to use this metric as a critical component of our *XModeScore* method (Borbulevych *et al.*, 2016 ▸). As detailed in Fu *et al.* (2011 ▸), the ligand-strain energy *E*_strain_ is computed as
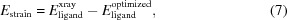
where 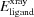 is the single point energy computed for the ligand X-ray geometry and 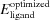 is the energy of the optimized ligand that corresponds to the local minimum.

When discussing ligand strain, it should be noted that it can be thought of as a combination of a number of different factors, as represented qualitatively by
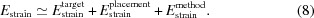
In this equation, 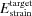 is the ‘natural’ or ‘target-induced’ strain associated with changes in ligand geometry/conformation owing to binding, 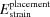 is the strain associated with initial ligand placement (*e.g.* docking) and 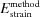 is the strain related to the underlying method or force field (restraints/CIF, functional or Hamiltonian) being used in the refinement. Ideally, 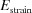 would equal 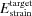. The *PHENIX*/*DivCon* plugin is primarily designed to address the 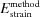 term through the replacement of inaccurate stereochemical restraints and approximate molecular-mechanics parameters with more accurate QM gradients which significantly reduce the method-induced ligand strain, as shown in our previous work (Borbulevych *et al.*, 2014 ▸). In order to address the 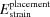 term in (8), additional side-chain sampling and/or ligand re-docking would need to be performed. These steps are beyond the scope of the present work, and the observed results are attributable to localized changes (for example improvements in bond lengths, torsions, rotations and translations) within the radius of convergence of the input conformation.

#### 2.3.2. Difference density as a measure of the accuracy of density around a ligand   

The conventional quality metric used to communicate agreement between the model and the X-ray (or neutron) density is the real-space correlation coefficient (RSCC; Brändén & Jones, 1990 ▸). However, in 2012 Tickle demonstrated that the RSCC correlates with both the accuracy and the precision of the structure model, and described a more sophisticated quality indicator, the real-space *Z* score of difference density (ZDD), which measures the accuracy of the model alone (Tickle, 2012 ▸; Borbulevych *et al.*, 2016 ▸). A detailed mathematical description of ZDD can be found in Borbulevych *et al.* (2016 ▸) and Tickle (2012 ▸), but briefly the *Z* score for a point difference density value is expressed by
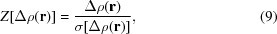
where σ[Δρ(**r**)] is the standard deviation of the difference density (*mF*_o_ − *DF*_c_) maps and corresponds to the random error of the model and is pure *precision*, while the *Z* score of the difference density is a measure of the residual, nonrandom error and is pure *accuracy*. In order to limit the impact of outliers or noise on the final value, while at the same time preserving information, we assume that the difference density *Z* values should approach a normal distribution of random errors with zero mean and unit standard deviation as the quality of the model, as measured by χ^2^, improves. The subset of values of *x* ^2^_(*i*)_ that maximize the probability *p*_max_ over *k* are summed,
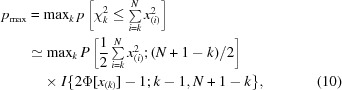
where the function *P* is the lower normalized gamma function representing the cumulative distribution function (CDF) of χ_*k*_^2^. The second function, *I*, is also computed as the complement in practice and is the normalized incomplete beta function (CDF of a normal-order statistic; Gibbons & Chakraborti, 2010 ▸) which accounts for the ‘multiple comparisons’ correction (Yuriev & Ramsland, 2013 ▸).

ZDD is evaluated as the two-tailed normal *Z* score corresponding to the maximal value *p*_max_ over *k* of the cumulative probability of χ_*k*_^2^ derived from (10),
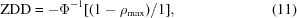
where the function Φ is the CDF of the normal distribution, 2Φ(|*Z*|) − 1 is the CDF of the half-normal distribution of the absolute value of a normal variate *Z*, and Φ^−1^ is the inverse function or the value of *Z* corresponding to a given probability. The set of negative density values, owing to incorrectly positioned atoms, yields ZDD−. Likewise, the set of positive density values, owing to missing atoms, yields ZDD+. The final ZDD is the maximum of the absolute values of ZDD− and ZDD+ as defined using
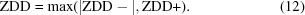
Thus, ZDD as used below is always positive and lower values correspond to a lower amount of residual difference density. Tickle (2012 ▸) provided further guidance to interpreting ZDD, such as a magnitude of over 3 indicates significant difference density peaks.

#### 2.3.3. Overall structure-quality metrics: *MolProbity* score and clashscore   

*MolProbity*, which is included as a module in *PHENIX*, is a software tool that includes several macromolecular model-validation metrics using multiple quality criteria (Chen *et al.*, 2010 ▸). The *MolProbity* score (MPScore) represents overall structure quality and is a logarithm-based score combining three key component metrics: clashscore, Ramachadran plot outliers (MacCallum *et al.*, 2009 ▸) and rotamer outliers (Hintze *et al.*, 2016 ▸; Lovell *et al.*, 2000 ▸). The lower the value of the MPScore, the better the quality of the model. In particular, an important component of the MPScore is the clashscore, which is the number of clashes per 1000 atoms; it is determined through nonbonded atom contacts derived using a rolling-probe algorithm employed by the program *Probe* (Word *et al.*, 1999 ▸). A clash occurs when the dot surface around one atom overlaps the dot surface around another by greater than 0.4 Å (Davis *et al.*, 2007 ▸). Generally, a chemically incorrect model will yield a high number of clashes (Chen *et al.*, 2010 ▸). Since the stereochemical restraint function does not explicitly include electrostatics and other nonbonded interactions for attraction and repulsion, while AMBER and PM6 do include these attractive, and in particular repulsive, effects, one would expect that clashscore should be a particularly indicative metric.

## 3. Results   

### 3.1. *R*-factor analysis   

As shown in Supplementary Table S2, the ONIOM method yields an average *R*_work_ of 0.177 ± 0.02 and an average *R*_free_ of 0.218 ± 0.02. Similarly, conventional *PHENIX* refinement produces averages of 0.171 ± 0.02 and 0.217 ± 0.02, respectively, and Region-QM refinement yields averages of 0.174 ± 0.02 and 0.218 ± 0.02, respectively. Together, these results show that the ONIOM methodology does not negatively impact the overall agreement between the experimental data and the atomic structure models.

### 3.2. Overall structure-quality metrics   

#### 3.2.1. *MolProbity*: Ramachandran and rotamer scores   

Fig. 3 ▸ depicts a histogram of MPScores for all 80 Astex structures involved in the current study, in which the average MPScore of ONIOM-refined structures is 1.23 ± 0.32 units. This average is lower (better) than the corresponding values for Region-QM (1.81 ± 0.32 units) and conventional (1.75 ± 0.34 units) refinements. Furthermore, unlike the Region-QM and conventional refinements, ONIOM refinement shows a bimodal distribution in which the first peak is at 0.75 units and covers about 50% of the population, and the second peak is at 1.6 units and coincides with peaks that are also observed for conventional and Region-QM data. This second peak has a long tail for these less sophisticated methods, with ∼25% of conventional and Region-QM structures distributed in the 2.0+ unit bin.

An analysis of the individual Ramachadran and rotamer components that comprise MPScore indicates that ONIOM refinement leads to models which exhibit improved statistics *versus* the models yielded by both conventional and Region-QM refinements. For example, when comparing conventional refinement and ONIOM refinement, the average percentage of Ramachadran plot outliers decreases from 0.40% to 0.26%, while the residue population in the favorable regions of the Ramachadran plot slightly increases from 96.46% to 96.90%. In over 91% of the cases studied ONIOM leads to models with a Ramachandran plot and rotamer angles that are as good or better when compared with those from conventional refinement, demonstrating that use of the ONIOM plugin does not break or otherwise damage the final model.

#### 3.2.2. *MolProbity*: clashscore   

While a portion of the observed improvement in the MPScore is attributable to improvements in the Ramachandran and rotamer components, the largest improvement is seen for the clashscore component. As shown in Table 1 ▸, for the 80 Astex models studied the average clashscore is 1.10 ± 0.41 units for the ONIOM models, which is 4.5–5.0-fold lower (better) than the average clashscores for the conventional (4.83 ± 1.2 units) and Region-QM (5.54 ± 1.6 units) models. The clashscore histogram (Fig. 4 ▸) shows a clear peak around 0.5 units which comprises 90% of the ONIOM models, while a peak representing both conventional and Region-QM model data is located around 3.5 units. Furthermore, around 50% of the data in the conventional and Region-QM histograms are found in the tails of the respective peaks and are distributed in histogram bins of 4.5+ units and above, while no ONIOM data are found in this range. This observation suggests that the ONIOM QM/MM method utilized in this study exhibits greater consistency over the range of structures studied *versus* the use of stereochemical restraints alone in an automated (high-throughput) regime with default *phenix.refine* settings. Furthermore, since the Region-QM and conventional refinements yield similar results for the bulk of the protein structure, this would suggest that much of the improvement in clashscore is attributable to the use of the QM/MM Hamiltonian on the entire structure.

**Figure 5:**
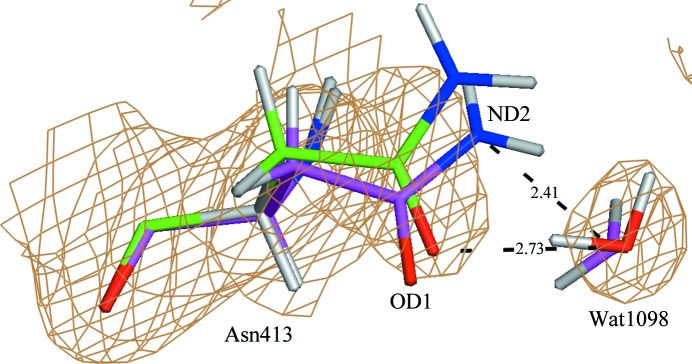
An example of resolving a bad clash between Asn413 and a water molecule in PDB entry 1sj0 after ONIOM refinement (green). The conventional refined structure is shown in magenta. The σ_A_-weighted 2*mF*_o_ − *DF*_c_ electron-density map is contoured at 1σ.

The crystal structure of human estrogen receptor α ligand-binding domain in complex with the antagonist ligand 4-D determined at 1.9 Å resolution (PDB entry 1sj0; Kim *et al.*, 2004 ▸) has been chosen as a representative example in order to demonstrate the sort of improvements that we have observed in treatment of the Astex Diverse Set with the QM/MM method. An initial clashscore for the deposited structure was calculated as 18.64 units. While all three refinements led to a noticeable reduction (improvement) in clashscore, ONIOM refinement exhibited the largest improvement, with a clashscore of 2.27 units compared with 9.06 units for conventional refinement and 13.09 units for Region-QM refinement. As shown in Supplementary Table S3, the poorer score of the conventional refinement is owing to the 36 bad clashes that remained after refinement (compared with 74 bad clashes in the originally downloaded file). 28 of those 36 clashes were not observed in the ONIOM model, and no additional clashes were introduced with ONIOM. Interestingly, owing to the addition of six clashes at the boundary of the buffer region, Region-QM refinement yielded a higher (worse) clashscore than both ONIOM and conventional refinement. Among the bad clashes observed after conventional refinement, ONIOM refinement leads to an average improvement of 0.25 ± 0.12 Å, while some significant short contacts were improved by as much as 0.65 Å. A notable example is depicted in Fig. 5 ▸, where the intermolecular distance between Asn413 ND and Wat1098 O in conventional refinement yields a clash distance of 2.41 Å, while ONIOM refinement yields a more reasonable 3.23 Å. Further, structural rearrangement in this region after ONIOM refinement is mostly attributable to the movement of the side chain of Asn413 (Fig. 5 ▸). This residue in the conventionally refined structure adopts an **m**-80° rotamer conformation, with the χ_1_ and χ_2_ torsion angles both being −84°. On the other hand, ONIOM refinement yields a χ_1_ angle in Asn413 which is increased by 15°, making this torsion angle (−69°) very close to the ideal value of −71° for the **m**-80° rotamer (Lovell *et al.*, 1999 ▸). Interestingly, this structural shift leads to the removal of both of the above-noted bad clashes and to an improvement in the Asn41 OD1–Wat1098 O bond distance (which approaches a typical hydrogen-bond distance). Specifically, when accompanied by the rotation of the Wat1098 water molecule depicted in Fig. 5 ▸, a hydrogen bond is indeed formed between Asn413 OD1 and Wat1098 O, as shown by the interatomic distance of 2.73 Å and the Wat1098 O–Wat1098 H1⋯Asn413 OD1 bond angle of 161° observed after ONIOM refinement.

#### 3.2.3. *MolProbity*: C^β^ deviations and r.m.s. bond and angle deviations   

In addition to the aforementioned Ramachandran, clashscore and rotamer components, for the sake of complete­ness the C^β^ deviations are also reported in Supplementary Table S2. Generally, C^β^ deviations are defined as abnormalities in bond-angle distributions around the C^β^ atom. Deviations larger than 0.25 Å typically indicate incompatibility between main-chain and side-chain conformations (Davis *et al.*, 2007 ▸). As indicated in Supplementary Table S2, the number of C^β^ deviations is similar in all three refinement types and over 90% of structures are free of this aberration. Furthermore, the average r.m.s.d. in bond length is the same for ONIOM (0.014 ± 0.002 Å), Region-QM (0.014 ± 0.002 Å) and conventional (0.013 ± 0.002 Å) refinements (Supplementary Table S2). However, the average r.m.s.d. in angles is slightly lower for conventional refinement (1.30 ± 0.20°) compared with QM-driven refinements (1.86 ± 0.20° for ONIOM and 1.53 ± 0.20° for Region-QM), suggesting greater variability in the QM and MM methods. This deviation is likely to be caused by different target bond angles in the AMBER functional together with the greater number of atom types in MM and the captured atom–atom interactions in both methods.

### 3.3. Ligand-quality metrics   

#### 3.3.1. Local ligand-strain energy   

**Figure 6:**
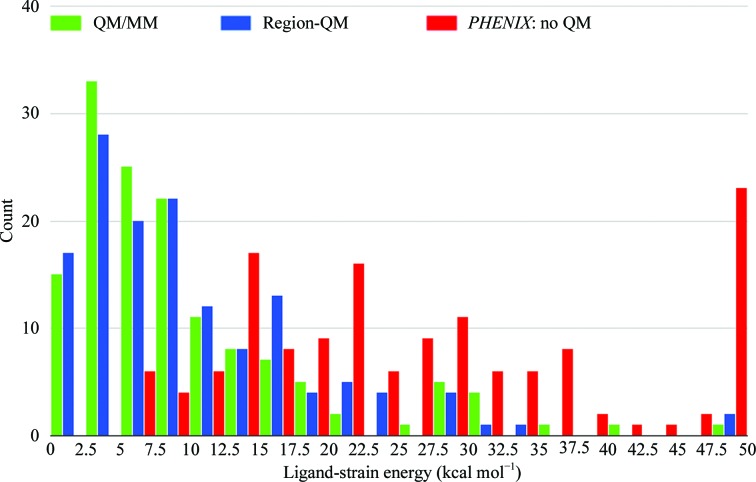
Histogram of ligand-strain energy distributions for 141 ligand instances from 80 Astex structures refined with three methods: ONIOM, Region-QM and conventional.

Ligand strain is a method to explore refined ligand structural models (Fu *et al.*, 2011 ▸; Janowski *et al.*, 2016 ▸; Mobley & Dill, 2009 ▸; Perola & Charifson, 2004 ▸), and ligand strain is a key metric which we have used previously to evaluate the quality of the region refinement (Borbulevych *et al.*, 2012 ▸, 2014 ▸). For the present study, we find that the average strain energies calculated over 141 ligands from 80 Astex structures are similar in ONIOM (9.95 ± 3.77 kcal mol^−1^) and Region-QM (10.49 ± 4.52 kcal mol^−1^) refinements. As shown in the ligand-strain histogram (Fig. 6 ▸), we also see similar distributions between both ONIOM refinement and Region-QM refinement in that both methods exhibit peaks around 3.0 kcal mol^−1^ which account for approximately three quarters of the models in the set. This is compared with conventional refinement using automatically generated CIFs, which yields a population of structures which are more evenly distributed in a broad range from 10 to 40 kcal mol^−1^ and ∼30% of the data are in the last bin of >50 kcal mol^−1^. This finding is consistent with our previous work, in which we demonstrated that QM refinement across a diverse population of structures yields a tighter strain energy range *versus* conventional methods (Borbulevych *et al.*, 2014 ▸). In addition to exhibiting a wider strain range, the average ligand-strain energy after conventional refinement of the Astex set is 35.64 ± 9.35 kcal mol^−1^ or about 3.5-fold higher than in the QM-driven refinements. This average improvement in strain energy is consistent with the 3.4-fold average improvement observed in Region-QM refinements in our previous study (Borbulevych *et al.*, 2014 ▸). While beyond the scope of the present work, which is focused on automated, high-throughput methods, arguably one could potentially manipulate these CIFs ‘by hand’ in order to yield ligand structures with lower strain energy or even which mimic the capture of atom–atom interactions (for example slightly elongated/shortened bond lengths, rotations *etc.*) automatically observed in QM/MM refinement. However, with over 80 species considered, these manipulations would come at a significant cost in investigator time with more opportunities for inclusion of investigator bias. Further, the success or failure of each structure would be much more greatly dependent on investigator proficiency.

**Figure 7:**
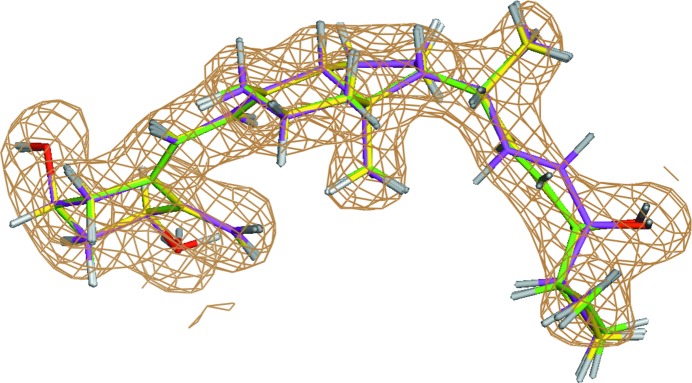
Superimposition of the ligand calcipotriol (ligand ID MC9) in PDB entry 1s19 refined with the ONIOM (green), Region-QM (yellow) and conventional (magenta) methods. The σ_A_-weighted 2*mF*_o_ − *DF*_c_ electron-density map is contoured at 1σ.

**Figure 8:**
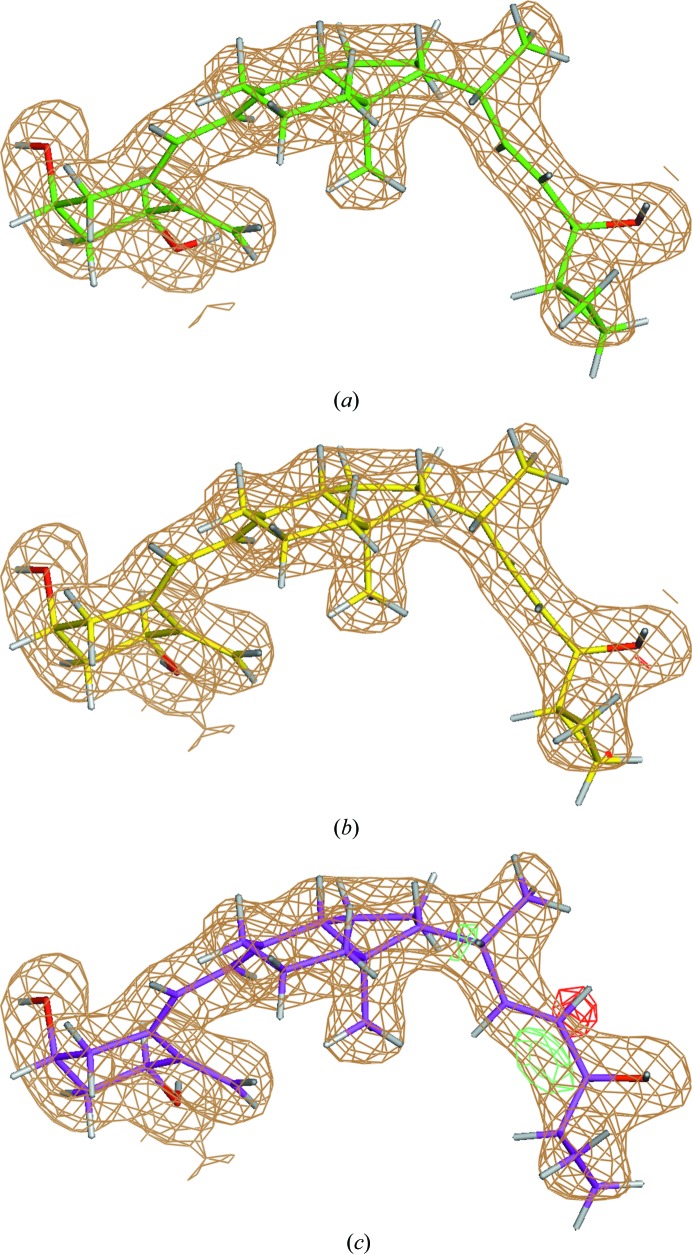
The σ_A_-weighted *mF*_o_ − *DF*_c_ difference electron-density map peaks around the ligand calcipotriol (ligand ID MC9) in PDB entry 1s19 refined with the ONIOM (*a*), Region-QM (*b*) and conventional (*c*) methods. The difference density is drawn at the 3σ level.

Refinement of the crystal structure of the vitamin D receptor (VDR) ligand-binding domain bound to calcipotriol (ligand ID MC9) determined at 2.1 Å resolution (PDB entry 1s19; Tocchini-Valentini *et al.*, 2004 ▸) is chosen as an illustrative example. Conventional refinement of PDB entry 1s19 leads to a strain energy of 28.62 kcal mol^−1^ for the ligand MC9 (Table 1 ▸). However, QM-driven refinement yields a ligand structural model in which ligand strains are 3.8–3.5-fold lower or 7.52 kcal mol^−1^ for ONIOM and 8.28 kcal mol^−1^ for Region-QM. Closer examination of the geometry of this ligand after the conventional and QM-driven refinements reveals that the key difference is related to the orientation of the hydroxyl­propene fragment at the junction with the cyclopropyl ring described by the torsion angle C22—C23—C24—C25, which is −35° for conventional refinement, −119° for ONIOM refinement and −128° for Region-QM refinement (Fig. 7 ▸). Further, the conventional model exhibits positive and negative density peaks (Fig. 8 ▸ *c*) which are not observed in the two QM-based refinements (Figs. 8 ▸ *a* and 8 ▸ *b*). These peaks generally indicate that the ligand conformation adopted is likely to be incorrectly placed within the density after conventional refinement.

#### 3.3.2. Ligand ZDD   

**Figure 9:**
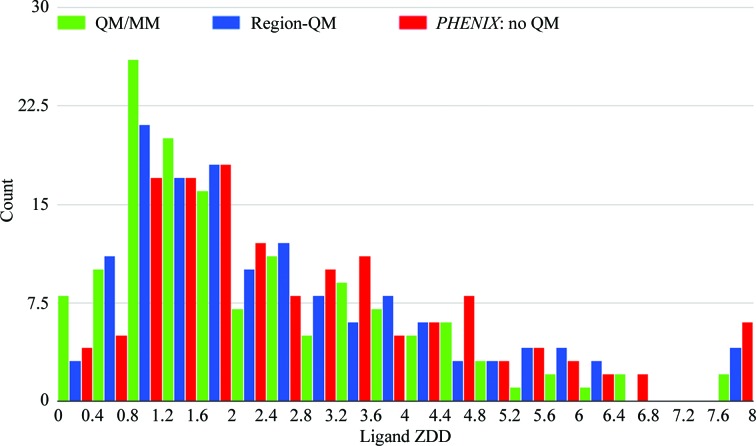
Histogram of ligand ZDD distributions for 141 ligand instances from 80 Astex structures refined with three methods: ONIOM, Region-QM and conventional.

The histogram for ZDD (Fig. 9 ▸) exhibits similar distributions for all three refinement types, with a rather broad peak at 1.4 units. However, the proportion of ONIOM- and Region-QM-refined models in the first three bins, which cover the range of values from 0 to 1.2 ZDD units, is higher than the number of conventional models in the same range. Thus, the average ZDD for the ligands in ONIOM-refined structures (2.3 ± 0.8 units) is slightly lower (better) than that after conventional refinement (2.9 ± 1.1 units). Region-QM refinement yields a set of models which are in the middle (2.6 ± 0.9 units) (Table 2 ▸). Overall, the ZDD distribution differs significantly from that observed in the ligand strain, and the square of the Pearson correlation coefficient (*R* ^2^) between ZDD and ligand strain is zero for all three refinements, demonstrating that these two metrics are uncorrelated.

## 4. Discussion   

Protein crystallography continues to play a central role in drug discovery as SBDD remains a critical technique for ligand design and optimization, high-throughput screening and often FDA approval (Blundell, 2017 ▸). However, the overall lackluster quality of ligands within deposited protein–ligand complexes raises serious concerns. Unfortunately, these errors in the ligand geometry, placement and protonation states often lead to the misperception of protein–ligand interactions and to problems in binding-mode determination, thus diminishing the relevance of such models for SBDD (Borbulevych *et al.*, 2012 ▸, 2014 ▸, 2016 ▸; Cooper *et al.*, 2011 ▸; Malde & Mark, 2011 ▸; Reynolds, 2014 ▸). These issues have been acknowledged (Debreczeni & Emsley, 2017 ▸), and the community has made significant methodological improvements in the generation of higher quality restraint (CIF) ligand dictionaries (Nicholls, 2017 ▸; Long *et al.*, 2017 ▸; Janowski *et al.*, 2016 ▸). However, these improvements still lead to a static dictionary file which is created for an isolated ligand without explicit consideration of the *in situ* impact of the protein and the ligand on one another. In our previous work (Borbulevych *et al.*, 2014 ▸), we introduced an approach for macromolecular refinement within the *PHENIX* package for Region-QM refinement. In this approach, the quality of the CIF is immaterial, and the entire user-defined region including both the ligand(s) and the active site(s) are treated as one QM system, thus capturing intermolecular interactions (for example electrostatics, charge transfer, polarization, dispersion and hydrogen bonding) at each refinement step. This work has led to significant improvements in ligand strain and ligand ZDD upon QM refinement. In cases where significant strain is still observed, manual building of the model may still be necessary to fix large model errors or rotamer outliers since any gradient-driven refinement cannot make changes beyond its radius of convergence.

The present study takes this improvement to macromolecular refinement further through the development and integration of a high-throughput and fully automated two-layer mixed QM/MM ONIOM module applied to the entire structure. With this fully automated approach, any user-chosen ligands, metal ions and cofactors, together with the surrounding residues, comprise a QM layer, while the rest of the atoms in the structure comprise the MM layer and interactions between the two layers are addressed. ONIOM refinement exhibits all benefits of the previously developed *DivCon* Region-QM refinement *versus* conventional refinement, as measured by ligand-strain energy and ligand ZDD (Table 1 ▸, Figs. 6 ▸ and 9 ▸), while at the same time showing marked improvements in overall structure quality as measured by MPScore (Table 1 ▸, Fig. 3 ▸). In particular, we observed an improvement in the clashscore component of MPScore by an average factor of 4.5–5.0 upon ONIOM refinement compared with both Region-QM and conventional *PHENIX* refinements (Table 1 ▸), demonstrating that ONIOM is able to correct bad clashes. The cause of these improvements can be explained when one considers how MM works. Specifically, since any residues outside the QM region are described at the MM level (in this case using the AMBER forcefield as implemented in *DivCon*), any reduction of unfavorable short clashes arises from the 6-12 Lennard–Jones potential for van der Waals interactions. Furthermore, the electrostatic interactions captured by the *q*_*i*_ *q*_*j*_/*r*_*ij*_ term of the AMBER functional also play an essential role, as shown by the example shown in Fig. 5 ▸. In this case, the bad clash between Asn413 ND2 and Wat1098 O that was found in the original structure, and that was not corrected by conventional and Region-QM refinement, was not only corrected by ONIOM but the interaction was also converted to an electrostatically favorable hydrogen bond.

When considering the distribution of ligand ZDD values (Table 1 ▸, Fig. 9 ▸), it is worth noting that ONIOM refinement leads to a smaller (better) average ZDD (2.29) when compared with the corresponding average for conventional refinement (2.94). However, this improvement is smaller in magnitude than that observed for ligand-strain energy. ZDD values generally correspond to the amount of difference density around the ligands (Borbulevych *et al.*, 2016 ▸; Tickle, 2012 ▸), and previously we have shown that ZDD is very sensitive to protomeric/tautomeric states (Borbulevych *et al.*, 2016 ▸) or ligand poses (Borbulevych & Westerhoff, 2018 ▸). However, in the present study the input PDB files including ligand states/positions were the same for all three types of refinement and therefore we would expect that the ZDD distributions would likewise be similar for those refinements (Fig. 9 ▸).

## 5. Conclusions   

Recently, numerous new programs and approaches to create high-quality ligand restraints have been published (Steiner & Tucker, 2017 ▸). These methods generally suffer from a critical, fundamental flaw in that they do not explicitly capture the *in situ* interactions between the protein and the ligand during refinement. In the present work, we demonstrate an entirely new methodology to perform X-ray refinement using the two-layer ONIOM method as implemented in the QuantumBio *DivCon* package. Using this concept, ligands and corresponding active-site residues are treated at the QM level, while the rest of molecule is represented using the MM functional. Both functionals are then combined to derive the ONIOM energy, and associated gradients, of the system. In the present work, the ONIOM approach for the X-ray refinement has been validated against 80 protein–ligand structures from the Astex Diverse Set using both *MolProbity* metrics and ligand-quality metrics. We established that ONIOM refinement excels in both sets of metrics, resulting in a superior overall quality of the protein–ligand model compared with conventional refinement. Combined with a fully automatic structure-preparation protocol and fast, convergent QM/MM calculations, we believe that the ONIOM refinement devised in this paper sets a new paradigm for fast, accurate and user-friendly macromolecular X-ray refinement.

## Supplementary Material

Supplementary Tables.. DOI: 10.1107/S2059798318012913/rr5160sup1.pdf

Final ONIOM files: PDB, MTZ, CIF.. DOI: 10.1107/S2059798318012913/rr5160sup2.gz

Final Region-QM files: PDB, MTZ, CIF.. DOI: 10.1107/S2059798318012913/rr5160sup3.gz

Final PHENIX files: PDB, MTZ, CIF.. DOI: 10.1107/S2059798318012913/rr5160sup4.gz

## Figures and Tables

**Table 1:** *MolProbity* statistics after ONIOM, Region-QM and conventional *PHENIX* refinements of 80 Astex PDB structures rota_out is the percentage of side chains with rotamer outliers, rama_fav is the percentage of amino acids in the ‘favored’ region of the Ramachandran plot and rama_iffy is the percentage of amino acids not in the ‘favored’ region of the Ramachandran plot.

		ONIOM					Region-QM					*PHENIX*: no QM				
PDB code	Res. (Å)	MPscore	Clashscore	rama_fav	rama_iffy	rota_out	MPscore	Clashscore	rama_fav	rama_iffy	rota_out	MPscore	Clashscore	rama_fav	rama_iffy	rota_out
1g9v	1.85	1.23	0.66	98.23	0	4.76	1.57	2.64	98.94	0	4.76	1.54	2.42	98.94	0	4.76
1gkc	2.30	1.47	1.82	97.43	0	3.56	1.77	6.28	96.78	0	1.98	1.54	3.24	96.78	0	1.98
1gpk	2.10	1.03	0.61	95.24	0.19	0.45	1.56	4.36	95.05	0.19	0.68	1.48	3.63	95.24	0.38	0.68
1hnn	2.30	1.75	1.47	97.33	0.38	9.22	2.52	9.55	95.99	0.38	9.46	2.48	8.08	95.99	0.38	10.17
1hp0	2.10	1.29	1.23	97.16	0	2.44	1.97	6.86	96.84	0	3.38	1.85	4.92	96.68	0	3.19
1hq2	1.25	0.74	0.77	98.72	0	0.00	0.83	1.15	99.36	0	0.00	0.74	0.77	99.36	0	0.00
1hvy	1.90	1.02	0.58	96.64	0.18	1.40	1.76	4.87	96.29	0.27	2.20	1.75	4.13	95.94	0.18	2.30
1hwi	2.16	1.15	0.30	96.60	0.20	2.62	1.74	3.61	96.60	0.20	3.03	1.75	3.05	96.21	0.13	3.36
1hww	1.87	0.89	0.86	97.33	0.20	0.44	1.49	5.36	96.74	0.40	0.44	1.36	4.81	97.43	0.40	0.55
1ia1	1.72	1.62	1.70	97.63	0.26	6.61	2.01	5.25	97.37	0.26	6.32	1.98	4.63	97.37	0.26	6.61
1ig3	1.90	0.98	0.25	96.55	0	1.60	1.66	5.32	96.55	0.20	1.60	1.65	5.44	96.75	0.41	1.60
1j3j	2.30	1.53	1.70	94.43	0.46	1.97	2.29	8.81	92.39	0.84	2.86	2.23	8.43	93.13	0.84	2.76
1jd0	1.50	0.82	1.10	98.25	0	0.43	1.09	2.70	97.86	0	0.43	1.04	2.33	97.86	0	0.43
1jje	1.29	1.39	0.29	95.65	0.23	4.24	2.24	4.86	95.42	0.92	7.69	2.12	3.86	95.42	0.46	6.63
1jla	2.50	1.66	0.89	95.53	0.53	5.83	2.78	11.98	91.28	1.28	7.81	2.74	10.52	91.38	0.96	8.04
1k3u	1.70	0.70	0.40	97.72	0.15	0.78	1.15	2.99	97.72	0.15	0.78	1.16	3.09	97.72	0.15	0.58
1ke5	2.00	0.96	1.97	98.55	0	0.00	1.49	6.99	97.45	0	0.41	1.43	6.99	97.82	0	0.82
1kzk	1.09	0.85	0.93	98.45	0	1.22	1.17	3.10	98.97	0	1.22	1.10	3.10	98.45	0	0.61
1l2s	1.94	0.60	0.27	98.02	0	0.89	1.04	2.10	97.74	0	0.71	1.05	2.01	97.74	0	1.07
1l7f	1.80	1.06	0.80	95.60	0	0.00	1.34	3.04	96.37	0	0.88	1.36	3.04	96.11	0	0.88
1lpz	2.41	1.53	0.45	95.41	0.35	5.33	2.33	5.36	95.05	0.71	8.20	2.19	4.25	95.05	0.71	6.97
1lrh	1.90	0.80	1.04	98.10	0	0.52	1.39	4.65	98.10	0	1.56	1.30	3.23	98.42	0	1.74
1meh	1.95	1.04	0.73	96.81	0.29	1.39	1.73	5.48	96.23	0.29	1.74	1.72	5.29	96.23	0.29	1.74
1mmv	2.00	0.87	0.60	97.52	0	1.24	1.82	6.20	96.16	0.12	1.92	1.73	4.86	96.16	0.12	1.92
1mzc	2.00	0.70	0.43	97.77	0	0.94	1.48	3.15	97.07	0.14	1.89	1.45	2.89	97.07	0.14	1.89
1n1m	2.50	2.23	3.74	92.34	1.17	5.98	2.83	14.82	91.52	1.66	7.13	2.76	13.06	92.07	1.45	7.13
1n2j	1.80	0.70	0.23	98.42	0	1.41	1.39	4.02	98.77	0	1.87	1.39	4.02	98.95	0	1.87
1n2v	2.10	1.52	1.21	96.49	0	3.95	2.31	10.74	95.14	0.27	3.62	2.18	8.49	95.68	0.27	3.62
1n46	2.20	1.99	3.83	97.27	0.42	7.93	2.67	11.60	95.81	1.05	11.42	2.55	9.69	96.44	1.05	11.42
1nav	2.48	1.64	1.26	93.93	0.40	3.24	2.12	5.03	93.52	0.40	3.70	2.10	4.53	93.12	0.40	3.70
1of1	1.95	0.89	0.94	98.34	0	1.40	1.59	6.05	97.19	0.50	1.40	1.55	5.11	97.35	0.50	1.60
1of6	2.10	1.82	1.89	95.96	0.51	6.06	2.09	3.71	95.34	0.51	6.28	2.05	3.59	95.60	0.44	6.15
1opk	1.80	0.88	0.28	97.32	0.22	1.53	1.61	2.35	96.20	0.45	2.81	1.51	1.94	96.42	0.45	2.56
1oq5	1.50	1.13	0	95.67	0	2.73	1.62	2.71	96.46	0	2.73	1.59	2.47	96.46	0	2.73
1owe	1.60	0.77	0.52	97.53	0	0.94	1.48	3.88	97.53	0	1.88	1.48	3.10	97.53	0	2.35
1oyt	1.67	1.03	1.30	97.45	0	1.21	1.21	3.04	97.45	0	0.40	1.13	2.39	97.45	0	0.40
1p2y	2.28	1.47	1.08	97.28	0	4.83	1.90	4.31	97.28	0	5.40	1.84	3.39	97.04	0	5.11
1p62	1.90	1.05	0.26	96.44	0	1.94	1.26	1.32	97.78	0	2.91	1.08	1.06	97.78	0	1.94
1q1g	2.02	1.18	1.27	97.37	0	1.86	1.81	6.46	96.54	0.07	2.02	1.64	3.97	96.68	0	2.10
1q41	2.10	1.05	1.00	96.73	0.74	1.16	1.64	3.92	95.83	1.19	1.66	1.51	3.01	95.83	1.19	1.50
1q4g	1.98	1.50	1.54	97.82	0.09	5.53	1.94	5.25	97.73	0	6.25	1.84	4.61	98.00	0	6.25
1r1h	1.95	1.98	3.76	97.12	0.14	7.44	2.32	9.21	97.12	0.43	7.60	2.30	8.41	96.97	0.43	7.60
1r55	1.59	0.71	0.63	98.01	0.5	0	1.39	4.43	97.01	0.50	0.00	1.37	4.11	97.01	0.5	0.00
1r58	1.90	1.34	1.04	94.28	1.91	1.58	1.92	8.13	94.01	1.91	1.26	1.99	8.13	94.01	1.91	1.58
1r9o	2.00	1.69	1.21	94.65	1.11	4.38	2.41	8.06	93.99	2.00	5.60	2.35	6.85	93.99	2	5.60
1s19	2.00	0.77	0	96.02	0	0.43	1.20	1.71	96.02	0	0.43	1.06	0.98	96.02	0	0.43
1s3v	1.80	1.57	1.95	97.28	0	4.17	2.18	4.87	95.65	0.54	6.55	2.13	4.87	96.20	1.09	6.55
1sg0	1.50	1.06	1.08	96.27	0	0.77	1.53	6.32	96.93	0	0.52	1.51	5.52	96.71	0	0.52
1sj0	1.90	1.70	2.27	97.07	0.84	5.07	2.61	13.09	95.82	0.84	8.29	2.36	9.06	96.65	0.84	7.37
1sq5	2.00	0.77	0.87	98.30	0.17	0.67	1.52	4.62	97.11	0.09	1.44	1.36	3.70	97.62	0.17	1.44
1t40	1.80	1.20	0.58	99.04	0	4.63	1.68	3.11	98.41	0.32	5.69	1.68	2.91	98.41	0	6.05
1t46	1.60	1.25	1.67	98.29	0	2.73	1.52	2.71	97.61	0	3.12	1.54	2.92	97.61	0	3.12
1t9b	2.20	0.94	1.32	98.38	0	1.26	1.24	4.03	98.29	0	1.16	1.18	3.14	98.63	0	1.26
1tow	2.00	1.37	0.96	96.12	0.78	2.61	1.62	4.78	96.90	0	1.74	1.77	3.82	96.90	0.78	3.48
1tt1	1.93	0.81	0.62	97.99	0	1.36	1.41	3.45	97.99	0	2.27	1.30	2.46	97.99	0	2.27
1tz8	1.85	0.97	1.61	98.45	0	1.21	1.36	4.53	98.23	0	1.51	1.24	3.95	98.23	0	1.21
1u1c	2.20	1.49	1.06	97.30	0.54	5.37	1.86	3.71	97.71	0.47	6.88	1.93	4.29	97.51	0.61	6.46
1u4d	2.10	1.32	1.20	96.84	0.20	2.45	2.08	5.75	96.25	0.40	4.68	1.96	4.31	96.25	0.59	4.45
1uml	2.50	2.44	3.40	92.51	2.31	12.83	3.00	12.52	91.35	2.31	14.47	3.03	12.16	90.78	2.31	15.46
1unl	2.20	1.71	0.77	95.77	0.69	7.66	2.56	6.74	93.59	1.26	10.05	2.56	6.88	93.48	1.14	9.67
1uou	2.11	1.54	1.85	97.69	0	4.92	2.13	8.31	97.69	0.23	6.46	2.12	8.78	97.92	0	6.77
1v0p	2.00	1.28	0.91	98.09	0.19	4.65	2.17	4.77	96.18	0.38	7.40	2.22	6.48	96.56	0.38	6.98
1v48	2.20	1.15	0.49	97.25	0.39	2.82	1.39	3.46	97.25	0	1.41	1.30	3.21	97.65	0.39	1.41
1v4s	2.30	1.42	1.00	95.52	0.45	2.60	2.37	7.99	92.60	0.67	4.17	2.31	6.71	93.50	0.67	4.69
1vcj	2.39	1.72	0.83	95.61	0	7.38	2.43	7.98	96.12	0	9.23	2.37	6.15	95.87	0.26	9.54
1w1p	2.10	0.98	1.34	97.78	0	1.24	1.58	5.06	97.17	0.10	1.61	1.54	4.54	97.48	0.1	1.86
1w2g	2.10	0.83	0.86	97.96	0.26	1.20	1.69	6.01	96.68	1.02	1.59	1.30	3.43	97.45	0.51	1.20
1x8x	2.00	0.70	0.59	99.38	0	0.75	1.12	2.95	99.06	0	1.12	1.12	2.95	99.06	0	1.12
1xm6	1.90	1.01	0.65	97.68	0.15	1.99	1.64	3.88	97.68	0.31	3.31	1.48	3.05	97.99	0.31	3.15
1xoq	1.83	0.61	0.28	98.29	0	1.01	1.07	1.77	98.44	0	1.52	0.97	1.40	98.60	0	1.35
1xoz	1.30	1.10	0.38	99.69	0	4.12	1.52	2.44	99.69	0	4.47	1.54	2.81	99.69	0	4.12
1y6b	2.10	0.88	0.46	97.27	0	1.29	1.24	3.68	98.05	0	1.29	1.47	4.38	97.66	0	1.72
1ygc	2.00	1.09	0.63	97.03	0	1.91	1.66	4.44	97.03	0	2.29	1.56	2.75	97.03	0	2.67
1yv3	1.99	0.57	0.19	98.27	0	0.91	1.08	1.68	98.12	0	1.64	1.15	1.86	97.98	0	1.82
1yvf	2.50	1.21	0.56	94.84	0.71	1.67	2.49	13.11	90.57	1.96	2.71	2.51	12.33	90.75	1.96	3.12
1ywr	1.90	2.3	3.08	95.18	0.9	13.76	2.88	11.43	95.18	0.9	18.79	2.87	10.88	94.88	0.6	18.46
1z95	1.80	1.53	3.03	98.29	0	3.70	1.81	6.82	98.72	0	3.70	1.85	6.82	98.72	0	4.17
2bm2	2.20	2.09	1.39	93.54	0	11.11	2.87	11.12	92.81	0.42	13.04	2.73	8.01	92.6	0.42	12.44
2br1	2.00	1.72	1.13	95.52	0.75	5.86	2.41	5.87	94.03	1.12	7.95	2.34	5.19	94.03	1.12	7.53
2bsm	2.05	1.35	0.3	94.17	0.97	2.82	1.83	2.73	91.75	0.97	2.26	1.79	2.73	92.72	0.97	2.26

**Table 2:** Strain-energy (kcal mol^−1^) and ZDD values for 141 ligands after ONIOM, Region-QM and conventional *PHENIX* refinements of 80 Astex PDB structures

			ONIOM		Region-QM		Conventional	
PDB code	Res. (Å)	Ligand	Strain energy	ZDD	Strain energy	ZDD	Strain energy	ZDD
1g9v	1.85	RQ3_A_801	5.11	2.5	4.74	2.1	27.01	2.1
1g9v		RQ3_C_802	3.65	3.0	4.29	2.9	31.72	3.5
1gkc	2.30	NFH_A_1448	10.36	1.0	11.78	0.9	22.10	1.1
1gkc		NFH_B_1449	10.80	4.6	10.35	4.7	27.53	3.3
1gpk	2.10	HUP_A_1540	2.61	1.1	3.87	0.3	10.13	0.4
1hnn	2.30	SKF_A_3001	5.48	1.5	9.07	1.2	12.84	0.8
1hnn		SKF_B_3002	8.43	3.1	9.03	3.9	15.67	4.0
1hp0	2.10	AD3_A_1315	17.65	5.9	15.83	5.6	25.79	5.9
1hp0		AD3_B_1316	15.42	2.4	14.32	2.9	18.03	2.6
1hq2	1.25	PH2_A_181	9.98	3.2	11.17	4.3	27.93	4.3
1hvy	1.90	D16_A_414	9.29	4.9	10.20	3.7	41.92	4.4
1hvy		D16_B_415	8.10	3.8	9.81	4.8	49.22	4.4
1hvy		D16_C_416	8.73	5.4	9.42	3.8	35.70	5.5
1hvy		D16_D_417	9.62	2.5	8.36	2.8	49.21	3.0
1hwi	2.16	115_A_2	6.14	1.5	5.58	0.5	22.29	0.8
1hwi		115_B_1	29.81	1.5	14.69	1.2	29.92	1.5
1hwi		115_C_4	10.86	1.5	15.65	2.5	31.13	2.0
1hwi		115_D_3	16.54	1.7	10.32	1.5	24.88	1.1
1hww	1.87	SWA_A_1103	29.64	2.4	16.36	0.9	13.07	0.1
1ia1	1.72	TQ3_A_194	2.01	1.2	1.65	1.4	9.99	3.0
1ia1		TQ3_B_196	2.57	1.7	2.92	1.7	10.87	2.4
1ig3	1.90	VIB_A_502	4.37	1.8	6.16	2.8	14.81	3.5
1ig3		VIB_B_501	2.72	4.4	4.78	5.3	13.38	6.0
1j3j	2.30	CP6_A_609	30.44	4.7	22.57	6.0	64.86	5.0
1j3j		CP6_B_709	1.07	1.6	1.71	1.4	89.56	8.8
1jd0	1.50	AZM_A_1400	6.23	6.4	5.58	7.7	37.34	6.5
1jd0		AZM_B_2401	12.73	4.4	14.09	7.8	34.54	4.5
1jje	1.29	BYS_A_250	28.44	2.8	29.37	4.1	31.76	3.5
1jje		BYS_B_250	31.08	4.0	32.20	5.1	46.87	4.4
1jla	2.50	TNK_A_999	68.96	1.3	71.30	2.7	187.39	2.6
1k3u	1.70	IAD_A_801	20.12	3.7	20.90	4.3	28.03	5.7
1ke5	2.00	LS1_A_299	11.98	2.5	8.84	2.5	28.62	4.5
1kzk	1.09	JE2_A_701	16.31	0.9	10.94	1.6	19.18	1.7
1l2s	1.94	STC_A_1115	4.27	1.6	6.71	2.3	11.07	2.8
1l2s		STC_B_2115	2.98	3.7	4.60	3.0	13.00	3.1
1l2s		STC_B_3115	8.30	13.3	6.07	13.9	29.20	15.4
1l7f	1.80	BCZ_A_801	8.52	1.5	9.04	1.8	21.23	1.9
1lpz	2.41	CMB_B_301	11.11	3.6	12.73	2.8	69.81	3.0
1lrh	1.90	NLA_A_5190	4.51	3.6	5.23	3.8	6.80	3.9
1lrh		NLA_B_6190	4.13	1.9	5.26	2.5	7.33	1.9
1lrh		NLA_C_7190	3.91	1.9	4.23	2.4	6.12	1.9
1lrh		NLA_D_8190	4.23	0.7	5.23	1.1	6.87	1.1
1meh	1.95	MOA_A_600	3.75	1.8	2.85	1.4	19.07	1.5
1mmv	2.00	3AR_A_1785	29.88	1.5	27.67	1.4	37.18	1.6
1mmv		3AR_B_2785	35.22	1.3	33.81	1.2	34.36	1.9
1mzc	2.00	BNE_B_1003	3.85	1.1	4.13	0.9	23.42	2.5
1n1m	2.50	A3M_A_954	4.90	1.6	9.72	1.8	14.36	0.8
1n1m		A3M_B_955	6.60	1.5	16.58	2.2	27.12	0.9
1n2j	1.80	PAF_A_1001	6.10	1.6	4.78	1.6	6.93	0.9
1n2j		PAF_B_1002	6.57	2.1	4.92	1.5	8.99	0.5
1n2v	2.10	BDI_A_900	10.50	1.2	15.59	1.4	13.29	1.4
1n46	2.20	PFA_A_462	31.06	0.1	23.01	0.6	68.42	0.9
1n46		PFA_B_463	30.63	0.7	27.54	1.2	72.57	0.8
1nav	2.48	IH5_A_600	7.37	0.5	9.33	1.1	39.41	2.0
1of1	1.95	SCT_A_400	2.86	1.1	2.64	0.9	16.10	1.5
1of1		SCT_B_500	5.31	0.7	4.49	1.0	15.90	1.3
1of6	2.10	DTY_A_1370	8.28	1.7	7.51	3.4	151.20	2.0
1of6		DTY_B_1370	10.02	0.7	8.94	2.1	149.66	2.1
1of6		DTY_C_1371	7.28	1.1	7.62	2.3	155.81	2.1
1of6		DTY_D_1370	11.18	0	8.19	0.8	146.43	0.2
1of6		DTY_E_1370	6.58	0.2	8.08	0.2	154.67	0.4
1of6		DTY_F_1370	8.00	0.0	8.40	1.0	163.72	0.9
1of6		DTY_G_1369	8.35	0.4	6.51	2.4	160.73	1.4
1of6		DTY_H_1369	9.36	0.8	9.67	1.6	150.23	1.1
1opk	1.80	P16_A_2	2.10	5.6	1.84	6.0	35.02	6.2
1oq5	1.50	CEL_A_701	11.81	3.2	15.60	3.7	19.44	4.2
1owe	1.60	675_A_1001	8.71	2.6	11.22	1.9	14.06	2.0
1oyt	1.67	FSN_H_501	8.40	3.5	8.85	3.4	27.29	4.2
1p2y	2.28	NCT_A_440	2.08	1.6	1.05	2.1	13.39	1.6
1p62	1.90	GEO_B_302	11.64	0.2	9.97	0.4	19.44	2.0
1q1g	2.02	MTI_A_301	15.92	3.2	15.04	3.4	32.53	3.9
1q1g		MTI_B_302	13.60	2.1	13.15	1.7	30.40	2.8
1q1g		MTI_C_303	15.61	3.5	14.22	2.9	29.91	5.5
1q1g		MTI_D_304	18.04	2.6	17.90	2.8	26.51	3.5
1q1g		MTI_E_305	14.46	1.1	18.81	0.7	31.12	1.9
1q1g		MTI_F_306	15.61	2.3	13.54	2.4	26.37	5.4
1q41	2.10	IXM_A_451	1.37	2.7	1.59	3.4	36.35	3.4
1q41		IXM_B_452	1.59	6.3	1.92	5.6	35.60	6.5
1q4g	1.98	BFL_A_701	2.55	3.2	3.97	5.2	6.53	3.9
1q4g		BFL_B_1701	2.47	6.5	3.76	6.2	7.92	5.6
1r1h	1.95	BIR_A_2001	13.81	1.0	17.32	1.9	33.90	1.7
1r55	1.59	097_A_518	21.70	1.5	16.20	1.6	29.71	2.0
1r58	1.90	AO5_A_501	41.38	3.9	62.41	3.9	68.73	2.9
1r9o	2.00	FLP_A_501	4.58	1.8	2.32	1.6	9.40	1.2
1s19	2.00	MC9_A_500	7.52	1.1	8.28	1.6	28.62	4.3
1s3v	1.80	TQD_A_187	8.60	0.6	9.10	0.8	29.76	1.4
1sg0	1.50	STL_A_501	2.55	10.2	2.88	10.1	12.51	12.0
1sg0		STL_B_502	4.41	4.8	5.96	5.6	14.94	4.5
1sj0	1.90	E4D_A_600	13.33	2.7	18.99	3.8	33.95	2.7
1sq5	2.00	PAU_A_6001	6.06	1.6	6.12	1.2	17.33	3.2
1sq5		PAU_B_6003	9.28	4.6	10.13	5.5	21.19	4.8
1sq5		PAU_C_6002	9.53	4.6	10.26	5.7	23.71	4.5
1sq5		PAU_D_6004	7.67	2.3	7.54	3.6	16.22	3.3
1t40	1.80	ID5_A_320	13.61	1.6	6.41	1.3	16.32	0.8
1t46	1.60	STI_A_3	16.69	3.5	17.06	4.3	59.53	3.2
1t9b	2.20	1CS_A_695	3.32	4.3	4.09	4.7	21.14	4.6
1t9b		1CS_B_1695	3.05	4.1	4.75	4.5	33.33	4.9
1tow	2.00	CRZ_A_501	7.25	1.1	3.74	0.7	10.99	0.5
1tt1	1.93	KAI_A_998	7.04	1.2	23.20	1.1	22.77	1.6
1tt1		KAI_B_999	6.31	1.1	22.03	0.8	22.88	1.2
1tz8	1.85	DES_B_128	5.09	3.2	3.98	2.7	62.82	13.9
1tz8		DES_C_129	0.24	3.8	0.82	4.3	56.87	9.1
1tz8		DES_D_128	1.11	0.8	0.28	0.7	46.17	10.0
1u1c	2.20	BAU_A_5400	7.88	4.1	13.09	4.2	22.15	3.5
1u1c		BAU_B_5011	8.83	1.3	15.44	2.2	21.01	0.8
1u1c		BAU_C_5021	4.66	2.4	18.31	2.7	22.46	3.0
1u1c		BAU_D_5031	7.33	2.4	20.52	2.5	22.11	2.2
1u1c		BAU_E_5041	7.62	1.1	11.21	0.5	18.81	1.2
1u1c		BAU_F_5051	5.87	0.7	17.19	1.0	23.16	1.2
1u4d	2.10	DBQ_A_398	5.44	0.1	5.66	0.4	21.99	0.1
1u4d		DBQ_B_401	5.46	0.8	3.54	1.2	18.50	1.8
1uml	2.50	FR4_A_1001	10.24	2.1	11.81	2.5	26.14	3.8
1unl	2.20	RRC_A_1293	25.22	3.2	27.55	3.2	83.26	4.0
1uou	2.11	CMU_A_1481	4.73	0.8	5.04	1.0	20.52	1.5
1v0p	2.00	PVB_A_1287	6.68	1.1	8.45	1.7	36.39	1.8
1v0p		PVB_B_1287	6.26	2.9	5.70	2.0	37.24	2.4
1v48	2.20	HA1_A_290	29.71	0.3	22.82	0.8	38.12	0.4
1v4s	2.30	MRK_A_501	2.99	2.1	2.79	2.0	27.23	2.4
1vcj	2.39	IBA_A_1	14.97	1.1	15.13	1.3	27.21	2.8
1w1p	2.10	GIO_A_1518	4.21	1.0	1.74	1.6	14.11	1.2
1w1p		GIO_B_1501	3.49	2.2	1.53	2.0	17.21	1.5
1w2g	2.10	THM_A_1210	3.03	1.0	7.16	1.1	10.95	1.5
1w2g		THM_B_1210	4.21	1.3	5.25	1.9	14.94	1.7
1x8x	2.00	TYR_A_952	14.47	0.1	4.77	0.1	101.28	0.1
1xm6	1.90	5RM_A_1003	0.71	0.4	1.32	0.6	12.15	0.9
1xm6		5RM_B_1003	2.67	1.0	2.22	0.4	15.49	1.4
1xoq	1.83	ROF_A_502	2.92	0.8	3.00	0.9	20.01	1.1
1xoq		ROF_B_501	2.55	1.3	2.97	1.1	21.02	1.4
1xoz	1.30	CIA_A_501	3.60	0.6	3.19	1.6	18.75	1.1
1y6b	2.10	AAX_A_201	6.88	4.9	6.89	5.4	19.13	5.5
1ygc	2.00	905_H_1	17.62	0.9	22.37	1.8	44.66	1.9
1yv3	1.99	BIT_A_800	3.51	2.8	2.75	3.2	12.91	2.7
1yvf	2.50	PH7_A_800	1.94	1.3	2.95	2.4	30.45	2.8
1ywr	1.90	LI9_A_361	17.66	4.1	21.03	4.8	52.45	3.9
1z95	1.80	198_A_501	5.73	1.3	6.55	1.1	28.01	3.3
2bm2	2.20	PM2_A_3211	1.43	1.4	2.27	1.4	14.88	1.8
2bm2		PM2_B_3211	1.87	0.9	1.96	1.1	21.68	2.3
2bm2		PM2_C_3211	1.56	1.0	1.38	1.4	12.97	1.9
2bm2		PM2_D_3211	1.40	1.8	1.41	1.1	13.31	1.7
2br1	2.00	PFP_A_1277	5.63	0.9	7.29	0.6	21.90	2.2
2bsm	2.05	BSM_A_1224	19.15	1.3	10.32	1.6	21.54	1.8
