# Supplementary material for: High-throughput quantum-mechanics/molecular-mechanics (ONIOM) macromolecular crystallographic refinement with PHENIX/DivCon: the impact of mixed Hamiltonian methods on ligand and protein structure
Source: Acta Crystallogr D Struct Biol. 2018 Oct 29;74(Pt 11):1063–77. doi: 10.1107/S2059798318012913 (PMC6213575; doi:10.1107/S2059798318012913)
Supplement: Supplementary file 1 [file d-74-01063-sup1.pdf]

STRUCTURAL
BIOLOGY

Volume 74 (2018)

Supporting information for article:

High-throughput QM/MM (ONIOM) macromolecular crystallographic refinement with *PHENIX*/*DivCon*: impact of mixed Hamiltonian methods on ligand and protein structure

Oleg Borbulevych, Roger I. Martin and Lance M. Westerhoff

Table S1 The PDB list of Astex structures involved in the present study.

1G9V 1GKC 1GPK 1HNN 1HP0 1HQ2 1HVY 1HWI 1HWW 1IA1 1IG3 1J3J 1JD0 1JJE 1JLA 1K3U 1KE5 1KZK 1L2S 1L7F 1LPZ 1LRH 1MEH 1MMV 1MZC 1N1M 1N2J 1N2V 1N46 1NAV 1OF1 1OF6 1OPK 1OQ5 1OWE 1OYT 1P2Y 1P62 1Q1G 1Q41 1Q4G 1R1H 1R55 1R58 1R9O 1S19 1S3V 1SG0 1SJ0 1SQ5 1T40 1T46 1T9B 1TOW 1TT1 1TZ8 1U1C 1U4D 1UML 1UNL 1UOU 1V0P 1V48 1V4S 1VCJ 1W1P 1W2G 1X8X 1XM6 1XOQ 1XOZ 1Y6B 1YGC 1YV3 1YVF 1YWR 1Z95 2BM2 2BR1 2BSM

Table S2 Bond and Angle RMS, Cβ deviations and R_{work}/R_{free} values after ONIOM , Region-QM and conventional PHENIX refinements of 80 Astex PDB structures.

	ONIOM				Region-QM				Phenix			
PDB ID	Cβ dev	RMS bonds	RMS angles	Rwork/Rfree	Cβ dev	RMS bonds	RMS angles	Rwork/Rfree	Cβ dev	RMS bonds	RMS angles	Rwork/Rfree
1G9V	0	0.016	1.9	0.1457/0.1759	0	0.017	1.58	0.1445/0.1759	0	0.016	1.45	0.1428/0.1775
1GKC	0	0.017	2.08	0.1718/0.2313	2	0.015	1.64	0.1791/0.2296	2	0.011	1.02	0.1698/0.2251
1GPK	0	0.014	1.76	0.1807/0.2164	0	0.013	1.35	0.1722/0.2160	0	0.012	1.16	0.1704/0.2125
1HNN	0	0.014	2.09	0.1971/0.2490	0	0.015	1.9	0.1999/0.2577	2	0.014	1.41	0.1958/0.2560
1HP0	0	0.013	1.77	0.1728/0.2360	0	0.013	1.46	0.1693/0.2464	0	0.012	1.09	0.1651/0.2439
1HQ2	0	0.012	1.65	0.1392/0.1625	0	0.010	1.38	0.1359/0.1593	0	0.008	1.17	0.1350/0.1566
1HVY	4	0.013	1.75	0.1826/0.2208	4	0.014	1.47	0.1784/0.2253	4	0.014	1.27	0.1707/0.2194
1HWI	0	0.013	1.81	0.1782/0.2108	0	0.014	1.42	0.1723/0.2091	0	0.013	1.29	0.1675/0.2163
1HWW	0	0.012	1.61	0.1580/0.1903	0	0.011	1.13	0.1443/0.1851	0	0.011	1.06	0.1430/0.1832
1IA1	0	0.014	2.06	0.1450/0.1868	0	0.016	1.97	0.1432/0.1838	0	0.014	1.52	0.1416/0.1825
1IG3	0	0.014	1.76	0.1829/0.2089	0	0.014	1.46	0.1796/0.2098	0	0.013	1.21	0.1779/0.2096
1J3J	1	0.013	1.98	0.1772/0.2155	0	0.018	1.75	0.1636/0.2197	0	0.016	1.48	0.1628/0.2174
1JD0	0	0.015	1.69	0.1667/0.1963	0	0.012	1.33	0.1619/0.1916	1	0.009	1.17	0.1607/0.1900
1JJE	0	0.016	1.89	0.2387/0.2854	1	0.013	1.67	0.2396/0.2915	0	0.012	1.19	0.2362/0.2874
1JLA	0	0.014	1.91	0.1953/0.2509	2	0.017	1.66	0.1890/0.2512	0	0.017	1.51	0.1869/0.2475
1K3U	0	0.012	1.65	0.1552/0.1805	0	0.010	1.22	0.1418/0.1767	0	0.010	1.09	0.1400/0.1737
1KE5	0	0.015	1.92	0.1514/0.2015	0	0.014	1.45	0.1537/0.2013	0	0.013	1.11	0.1522/0.1993
1KZK	0	0.015	1.6	0.1896/0.2107	0	0.013	1.33	0.1893/0.2077	0	0.009	1	0.1871/0.2081
1L2S	0	0.013	1.64	0.1571/0.1899	0	0.012	1.31	0.1547/0.1920	0	0.011	1.03	0.1512/0.1894
1L7F	0	0.014	1.65	0.1416/0.1737	0	0.012	1.38	0.1353/0.1709	0	0.010	1.13	0.1324/0.1680
1LPZ	0	0.014	1.97	0.1460/0.2226	0	0.014	1.55	0.1452/0.2266	0	0.012	1.12	0.1420/0.2251
1LRH	0	0.015	1.73	0.1819/0.2241	1	0.014	1.82	0.1804/0.2291	1	0.011	1.24	0.1759/0.2243
1MEH	0	0.014	1.78	0.2155/0.2335	0	0.015	1.34	0.2094/0.2346	0	0.013	1.08	0.2080/0.2337
1MMV	0	0.014	1.78	0.1990/0.2366	0	0.017	1.4	0.1923/0.2419	0	0.014	1.29	0.1898/0.2401
1MZC	0	0.013	1.68	0.1514/0.1810	0	0.012	1.17	0.1436/0.1796	0	0.012	1.09	0.1417/0.1775
1N1M	0	0.014	1.93	0.1817/0.2434	0	0.015	1.48	0.1671/0.2458	0	0.015	1.37	0.1635/0.2431
1N2J	2	0.012	1.62	0.1701/0.2015	2	0.013	1.49	0.1703/0.1964	2	0.012	1.29	0.1663/0.1960

1N2V	0	0.013	1.88	0.1620/0.2150	0	0.014	1.5	0.1583/0.2154	0	0.013	1.2	0.1563/0.2131
1N46	0	0.015	2.03	0.1821/0.2441	0	0.019	1.99	0.1815/0.2488	0	0.017	1.53	0.1777/0.2482
1NAV	0	0.017	2.31	0.2073/0.2526	0	0.015	1.78	0.2150/0.2517	0	0.015	1.33	0.2096/0.2516
1OF1	0	0.013	1.72	0.1689/0.2008	0	0.013	1.45	0.1656/0.2051	0	0.011	1.1	0.1629/0.2014
1OF6	2	0.014	2	0.1895/0.2339	0	0.017	1.61	0.1885/0.2361	8	0.018	1.36	0.1846/0.2332
1OPK	0	0.013	1.7	0.1717/0.2051	0	0.012	1.41	0.1710/0.2046	0	0.012	1.12	0.1691/0.2041
1OQ5	0	0.013	1.73	0.1598/0.1864	0	0.012	1.44	0.1584/0.1880	0	0.010	1.15	0.1573/0.1863
1OWE	0	0.017	1.84	0.1721/0.2019	0	0.015	1.45	0.1726/0.2018	0	0.013	1.24	0.1719/0.2010
1OYT	0	0.014	1.8	0.1628/0.1905	0	0.012	1.45	0.1559/0.1868	0	0.010	1.18	0.1527/0.1851
1P2Y	0	0.016	2.01	0.1823/0.2436	0	0.016	1.62	0.1832/0.2446	0	0.017	1.47	0.1821/0.2454
1P62	0	0.017	1.88	0.1668/0.2069	0	0.014	1.57	0.1685/0.2070	0	0.012	1.15	0.1672/0.2060
1Q1G	0	0.013	1.58	0.1945/0.2275	0	0.015	1.41	0.1893/0.2287	1	0.014	1.16	0.1816/0.2253
1Q41	0	0.014	1.71	0.2009/0.2199	0	0.014	1.34	0.1994/0.2209	0	0.013	1.14	0.1968/0.2180
1Q4G	0	0.015	1.93	0.1858/0.1970	0	0.016	1.5	0.1785/0.1984	0	0.015	1.39	0.1771/0.1952
1R1H	0	0.014	2.01	0.2001/0.2510	0	0.014	1.35	0.1910/0.2511	0	0.014	1.22	0.1898/0.2499
1R55	0	0.014	1.92	0.1650/0.1900	0	0.013	1.66	0.1661/0.1918	0	0.011	1.42	0.1656/0.1892
1R58	0	0.014	1.88	0.1857/0.2284	0	0.013	1.38	0.1840/0.2303	0	0.012	1.15	0.1816/0.2294
1R9O	1	0.015	1.9	0.1715/0.2076	0	0.017	1.57	0.1694/0.2053	1	0.017	1.48	0.1668/0.2063
1S19	0	0.014	1.83	0.1499/0.1905	0	0.013	1.57	0.1551/0.1940	0	0.012	1.04	0.1502/0.1895
1S3V	0	0.017	2.24	0.1539/0.1974	0	0.016	1.83	0.1570/0.1985	0	0.015	1.42	0.1560/0.1966
1SG0	0	0.015	1.85	0.1823/0.2075	0	0.012	1.55	0.1810/0.2102	0	0.010	1.37	0.1783/0.2057
1SJ0	0	0.016	2.17	0.1914/0.2615	0	0.015	1.95	0.1934/0.2576	0	0.014	1.33	0.1889/0.2543
1SQ5	0	0.013	1.84	0.1975/0.2312	0	0.014	1.45	0.1883/0.2347	0	0.013	1.23	0.1821/0.2310
1T40	0	0.015	1.86	0.1339/0.1692	0	0.014	1.63	0.1340/0.1698	0	0.013	1.29	0.1321/0.1675
1T46	0	0.013	1.81	0.1941/0.2259	0	0.012	1.51	0.1924/0.2261	0	0.011	1.12	0.1901/0.2242
1T9B	0	0.012	1.68	0.1588/0.1880	0	0.013	1.19	0.1427/0.1807	0	0.012	1.08	0.1372/0.1765
1TOW	1	0.018	2.24	0.1502/0.2180	0	0.015	1.76	0.1630/0.2076	0	0.014	1.12	0.1546/0.2203
1TT1	0	0.013	1.67	0.1595/0.1953	0	0.014	1.44	0.1544/0.1930	0	0.013	1.14	0.1523/0.1897
1TZ8	0	0.014	1.69	0.1844/0.2078	0	0.011	1.32	0.1822/0.2091	0	0.011	1.04	0.1821/0.2089
1U1C	0	0.013	1.75	0.2049/0.2471	0	0.015	1.21	0.1943/0.2444	2	0.014	1.15	0.1796/0.2394
1U4D	0	0.014	1.88	0.1858/0.2302	0	0.016	1.67	0.1841/0.2318	0	0.015	1.32	0.1817/0.2315
1UML	2	0.016	2.47	0.1729/0.2369	1	0.017	1.97	0.1802/0.2391	0	0.017	1.65	0.1720/0.2306
1UNL	0	0.013	1.81	0.2077/0.2458	0	0.016	1.45	0.1960/0.2458	0	0.016	1.29	0.1949/0.2460
1UOU	0	0.013	2.02	0.1702/0.2423	0	0.015	1.7	0.1718/0.2505	0	0.014	1.38	0.1694/0.2498

1V0P	0	0.014	1.8	0.1916/0.2485	0	0.016	1.64	0.1886/0.2530	0	0.014	1.25	0.1860/0.2508
1V48	0	0.016	2.02	0.1563/0.2267	0	0.015	1.78	0.1595/0.2339	0	0.014	1.22	0.1558/0.2292
1V4S	0	0.013	1.87	0.2099/0.2557	0	0.015	1.49	0.2048/0.2549	0	0.015	1.29	0.2031/0.2544
1VCJ	0	0.015	2.07	0.1613/0.2223	0	0.015	1.69	0.1669/0.2282	0	0.014	1.31	0.1615/0.2246
1W1P	0	0.012	1.61	0.1907/0.2255	0	0.013	1.39	0.1807/0.2419	0	0.013	1.21	0.1776/0.2403
1W2G	0	0.013	1.79	0.1827/0.2310	0	0.014	1.73	0.1874/0.2269	0	0.011	1.17	0.1807/0.2316
1X8X	0	0.014	1.71	0.1569/0.1971	0	0.013	1.4	0.1570/0.1994	0	0.012	1.01	0.1558/0.1975
1XM6	0	0.014	1.76	0.2001/0.2300	0	0.013	1.34	0.1973/0.2313	0	0.012	1.09	0.1950/0.2301
1XOQ	0	0.013	1.66	0.1719/0.2040	0	0.012	1.26	0.1677/0.2061	0	0.010	1.03	0.1651/0.2051
1XOZ	0	0.012	1.61	0.1836/0.2106	0	0.011	1.24	0.1822/0.2116	0	0.010	1.08	0.1809/0.2106
1Y6B	0	0.016	1.93	0.1877/0.2292	0	0.015	1.55	0.1872/0.2206	0	0.014	1.22	0.1859/0.2197
1YGC	0	0.014	1.84	0.1632/0.1777	0	0.014	1.54	0.1620/0.1796	0	0.012	1.23	0.1590/0.1760
1YV3	0	0.017	1.85	0.1653/0.1973	0	0.012	1.3	0.1579/0.1974	0	0.012	1.16	0.1572/0.1969
1YVF	0	0.013	1.84	0.2139/0.2552	0	0.015	1.56	0.2081/0.2589	0	0.014	1.34	0.2059/0.2547
1YWR	1	0.015	2.07	0.2148/0.2617	0	0.014	1.71	0.2125/0.2580	0	0.014	1.39	0.2106/0.2569
1Z95	0	0.015	2	0.1934/0.2383	0	0.015	1.65	0.1945/0.2397	0	0.014	1.22	0.1941/0.2397
2BM2	0	0.015	2.02	0.1857/0.2359	0	0.015	1.65	0.1792/0.2392	0	0.014	1.32	0.1745/0.2389
2BR1	0	0.015	1.98	0.1551/0.2255	0	0.014	1.64	0.1564/0.2286	0	0.014	1.29	0.1552/0.2272
2BSM	0	0.016	2.04	0.1717/0.2305	0	0.015	1.74	0.1745/0.2369	0	0.014	1.25	0.1713/0.2338

Table S3 Bad clashes (Å) in the structure 1SJ0 found after conventional PHENIX refinement and corresponding contact distances resulted from ONIOM refinement.

The difference (Å) between the two sets is also calculated. Fixed clashes in ONIOM refinement are shown in bold.

Contact	PHENIX	ONIOM	Difference
OE2 Glu542 ... O Wat1121	2.06	**2.73**	0.67
HB3 Asn348 ... O Wat1093	1.92	**2.21**	0.29
NZ Lys467 ... O Wat1079	2.27	**2.92**	0.65
HG Leu310 ... HB2 Gln314	1.79	**2.08**	0.29
HE1 Met342 ... O Gln414	1.98	2.23	0.25
OG1 Thr311 ... HG3 Gln314	2.00	**2.26**	0.26
HB3 Cys381 ... O Wat1086	1.97	**2.3**	0.33
O Leu454 ... HG22 Val458	2.01	2.22	0.21
HB3 Arg412 ... O Wat1061	2.03	**2.49**	0.46
HA Gln498 ... CD2 His501	2.43	**2.51**	0.08
O Asn455 ... HG23 Val458	2.08	**2.37**	0.29
ND2 Asn413 ... O Wat1098	2.41	**3.23**	0.82
HG Leu539 ... CE Met543	2.44	**2.55**	0.11
OD2 Asp374 ... OE1 Glu471	2.32	**2.58**	0.26
CD2 Leu308 ... HB3 Arg477	2.45	**2.56**	0.11
OG Ser463 ... N Ser464	2.49	**3.05**	0.56
CZ2 Trp383 ... HE1 Met522	2.5	**2.8**	0.3
CG1 Val418 ... HE2 Met421	2.45	2.86	0.41
O Val446 ... O Wat1020	2.21	**2.57**	0.36
HD13 Ile358 ... HD13 Leu379	1.99	2.12	0.13
SD Met315 ... HG2 Pro365	2.54	**2.7**	0.16
HD22 Leu308 ... HE Arg477	1.82	1.81	-0.01
O Gly344 ... HB2 Asn348	2.15	**2.25**	0.1
HD3 Lys529 ... N Cys530	2.3	**2.58**	0.28
ND2 Asn348 ... O Wat1087	2.5	**3.19**	0.69
CD1 Tyr459 ... N Tyr459	2.86	**3.07**	0.21

H24 E4D600 ... S11 E4D600	2.55	2.52	-0.03
HB3 Tyr331 ... HD21 Leu345	2.01	**2.06**	0.05
CE2 Phe435 ... HG21 Ile510	2.53	2.52	-0.01
O Tyr526 ... CB Ser527	2.67	**2.95**	0.28
HD13 Leu403 ... HD13 Leu409	2.00	**2.11**	0.11
HG Leu539 ... HE2 Met543	2.03	**2.07**	0.04
CE2 Tyr328 ... HB2 Pro406	2.55	2.45	-0.1
HB3 Met490 ... HD12 Leu495	1.99	**2.1**	0.11
HD3 Lys401 ... HG Leu409	1.99	**2.13**	0.14
SD Met342 ... HD13 Leu410	2.61	**2.68**	0.07
